# Analysis of multi-level spatial data reveals strong synchrony in seasonal influenza epidemics across Norway, Sweden, and Denmark

**DOI:** 10.1371/journal.pone.0197519

**Published:** 2018-05-17

**Authors:** Sinead E. Morris, Birgitte Freiesleben de Blasio, Cécile Viboud, Amy Wesolowski, Ottar N. Bjørnstad, Bryan T. Grenfell

**Affiliations:** 1 Department of Ecology and Evolutionary Biology, Princeton University, Princeton, NJ, United States of America; 2 Department of Biostatistics, Oslo Centre for Biostatistics and Epidemiology, Institute of Basic Medical Sciences, University of Oslo, Oslo, Norway; 3 Department of Infectious Disease Epidemiology and Modelling, Norwegian Institute of Public Health, Oslo, Norway; 4 Fogarty International Center, National Institutes of Health, Bethesda, MD, United States of America; 5 Department of Epidemiology, Johns Hopkins Bloomberg School of Public Health, Baltimore, MD, United States of America; 6 Department of Biology, Pennsylvania State University, University Park, PA, United States of America; 7 Center for Infectious Disease Dynamics, Pennsylvania State University, University Park, PA, United States of America; University of Malaya, MALAYSIA

## Abstract

Population structure, spatial diffusion, and climatic conditions mediate the spatiotemporal spread of seasonal influenza in temperate regions. However, much of our knowledge of these dynamics stems from a few well-studied countries, such as the United States (US), and the extent to which this applies in different demographic and climatic environments is not fully understood. Using novel data from Norway, Sweden, and Denmark, we applied wavelet analysis and non-parametric spatial statistics to explore the spatiotemporal dynamics of influenza transmission at regional and international scales. We found the timing and amplitude of epidemics were highly synchronized both within and between countries, despite the geographical isolation of many areas in our study. Within Norway, this synchrony was most strongly modulated by population size, confirming previous findings that hierarchical spread between larger populations underlies seasonal influenza dynamics at regional levels. However, we found no such association when comparing across countries, suggesting that other factors become important at the international scale. Finally, to frame our results within a wider global context, we compared our findings from Norway to those from the US. After correcting for differences in geographic scale, we unexpectedly found higher levels of synchrony in Norway, despite its smaller population size. We hypothesize that this greater synchrony may be driven by more favorable and spatially uniform climatic conditions, although there are other likely factors we were unable to consider (such as reduced variation in school term times and differences in population movements). Overall, our results highlight the importance of comparing influenza spread at different spatial scales and across diverse geographic regions in order to better understand the complex mechanisms underlying disease dynamics.

## Introduction

Influenza is a respiratory infection of serious public health concern, with over three million cases of severe influenza-associated illness reported globally each year [1]. In temperate climates, epidemics occur each winter and leave signatures of spatial spread that are still not fully understood [2–4]. A rich literature from countries with historically available data, such as the United States (US) and France, associates such signatures with spatial distributions of population density, specific humidity conditions, and human movements [5–9]. However, to understand the true extent of these associations, analyses must be extrapolated across a greater range of demographic and climatic environments.

Norway is situated in Northern Europe, a temperate region near the Arctic Circle, and provides a novel environment to explore spatial patterns of influenza spread. Firstly, Norway is the longest country in Europe in terms of latitudinal range, with an extremely heterogeneous population distribution. Over 80% of inhabitants live in urban areas around the south and central coasts, whereas the northern regions are more sparsely populated and geographically isolated. One may therefore expect a strong spatial signature in influenza epidemics, driven by preferential spread between large and well-connected southern cities such as Oslo and Bergen [5].

On the other hand, underlying climatic variables, such as humidity and temperature, may modify these patterns in unexpected ways [10–13]. For example, low levels of specific humidity (or vapor pressure) promote increased survival of influenza virus, and modulate spatial dynamics in regions with diverse climatic environments such as the US [6, 11]. However, climatic conditions in Norway are more spatially homogeneous, with continental and temperate conditions dominating most populated regions of the mainland [14]. Norway therefore provides an ideal setting to explore how interactions between competing demographic and climatic distributions impact the spatiotemporal spread of influenza.

In addition to influenza spread within Norway, fluid population movements between neighboring countries underscore the importance of considering spread across a wider geographic scale [15]. For instance, high levels of commuting across the Swedish border may facilitate the importation of new seasonal strains and substantially alter local patterns of transmission in Norway. Across mainland Europe, previous studies have described eastward waves of influenza that bear hallmarks of a simple spatial diffusion process [16, 17]. However, demographic and climatic covariates were not included in these analyses, and so the underlying mechanisms remain unclear. Furthermore, these studies were limited in their use of national-level data, which may dilute patterns of spread at finer spatial resolutions. Integrating subnational data from Norway, Sweden, and Denmark with new demographic and climatic information will thus enable further exploration of the factors mediating influenza spread across national borders.

In this paper we first explore patterns of influenza spread in Norway using spatially rich data from the national sentinel surveillance system. We then investigate the role of geographic, demographic, and climatic factors in mediating these patterns, and incorporate data from Sweden and Denmark to extrapolate our findings across a wider geographic scale. Finally, we use the US as a demographically and climatically diverse temperate region with which to assess the generalizability of our results. To address discrepancies in geographic scale between Norway and the US, we develop a novel approach of partitioning the latter into geographical transects of comparable size to Norway. Our results highlight the importance of comparing influenza spread across different geographic regions if the dynamics of this pervasive disease are to be fully understood.

## Materials and methods

### Data

#### Epidemiological records

Influenza data for Norway were obtained from the Norwegian sentinel surveillance system (Norwegian Institute of Public Health) and contain weekly reports of influenza-like illness (ILI) cases for 16 consecutive influenza seasons, from 1998–2014. Such sentinel data have been shown to provide reliable estimates of influenza activity across Norway and the wider European region [17]. ILI cases are diagnosed by 201 general practitioner (GP) offices across Norway according to the International Classification of Primary Care (ICPC) code R80. GPs are required by law to submit weekly reports, and are spatially distributed according to geographic location, population size, and reporting frequency [18, 19]. Each office is identified by a unique 6-digit code that indicates the municipality and county in which it is located. Overall, the surveillance system represents approximately 16% of the Norwegian population and is assumed to be geographically representative [19].

**Fig 1:**
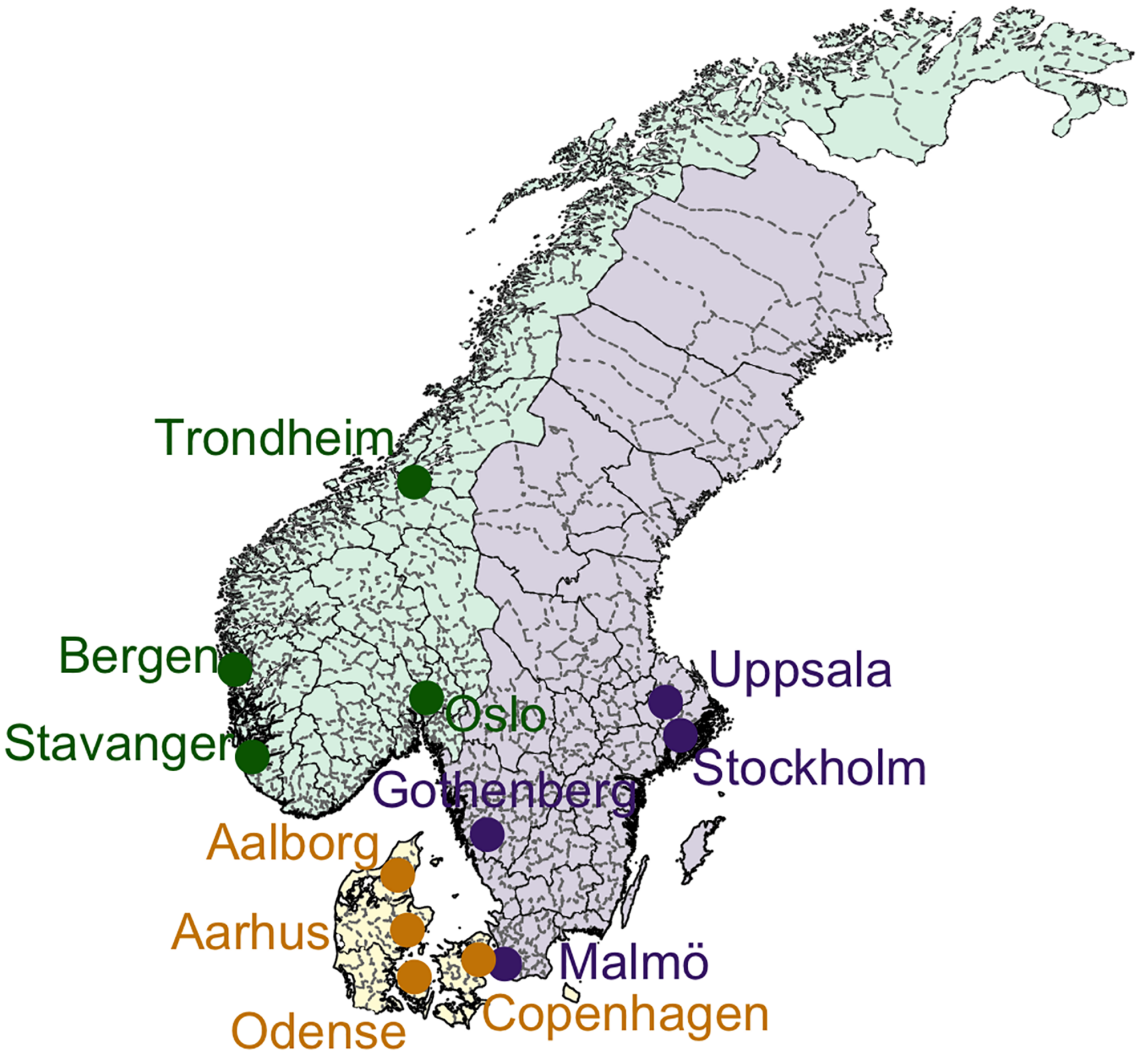
Population centers. The four most populated cities in Norway (green), Sweden (purple), and Denmark (yellow) are marked on the map. County borders are depicted by solid black lines and municipality borders by grey dotted lines.

The Danish data were obtained from the Danish sentinel surveillance system (Statens Serum Institut) and contain weekly ILI cases reported by approximately 140 GPs from 2001–2014. Similar to Norway, the data indicate the municipality and region (equivalent to Norwegian counties) in which each GP is located. The Swedish data were obtained from the Swedish virological surveillance system (Public Health Agency of Sweden) and consist of weekly positive confirmed cases of influenza from 2001–2015. These are reported by up to 27 laboratories across the country, and contain information on the county in which each laboratory is located. The majority of samples are processed in the county in which the corresponding case originated, and thus the data are assumed to be spatially representative. To coincide with peak influenza transmission, reporting in all three countries typically extends from week 40 of each year to week 20 of the following year. Notable exceptions were the pandemic seasons of 2009/10 and 2010/11 for which reporting continued year-round. The spatial boundaries of all municipalities and counties within each country are depicted in Fig 1.

The US data were obtained from IMS Health (Intercontinental Marketing Statistics; now known as IQVIA) electronic medical claims records and have been described previously [8, 20, 21]. Briefly, the data contain standardized weekly ILI cases from outpatient physician visits across the country, and cover approximately 70% of the US population [20]. In contrast to the Norwegian, Swedish, and Danish data, sampling was conducted year-round from 2002–2009. The data were aggregated by city using the zip code of each physician office, and only cities in the 48 contiguous states with over 100,000 inhabitants were extracted for analysis [21]. The resulting data from 329 cities have been shown to accurately capture the seasonal dynamics of influenza at fine spatial resolutions [20].

#### Demographic, geographic, and climatic data

In brief, for Norwegian municipalities we obtained publicly available information on population sizes, latitude and longitude coordinates, altitude landscapes, flow of passengers traveling between Norwegian airports, and daily temperature and specific humidity records (S1 Fig). We also obtained population sizes, latitude and longitude coordinates, temperature, and humidity data for counties in Norway, Sweden, and Denmark (S2 Fig), and cities in the US. Further details of these data and where they can be obtained are given in S1 Text.

### Analysis

To explore fine-scale spatiotemporal patterns of transmission within Norway, the ILI incidence data were aggregated at the municipality-level. Although weekly ILI records are often normalized by the weekly number of GP visits, we excluded this step as the latter data were unavailable for the first period of our study (1998–2003). Comparative analyses of the ILI and normalized ILI time-series from 2004–2014 produced qualitatively similar results and so we do not expect this omission to impact our conclusions. Of the 428 municipalities in Norway, 165 were originally represented in the data. To account for low sample sizes we removed 29 that failed a conservative Box-Pierce white noise test or had more than two seasons with less than ten total cases reported (S3 Fig) [22]. The remaining 136 ILI time-series were used for analysis of spatial synchrony in the amplitude (or magnitude) of influenza epidemics (S4 Fig).

To analyze synchrony in epidemic timing, wavelet phase-angles were also reconstructed from the 136 ILI time-series. Following standard procedures, each series was first padded with zeros to the nearest power of two, then log-transformed and standardized to reduce variability and noise obscuring the underlying periodic features [5, 23]. The standardized series were then decomposed into periodic signals using a Morlet wavelet function within the ‘WaveletComp’ library in R [24]. Finally, phase-angle trajectories were reconstructed from the dominant annual period of each decomposed series and used for the synchrony analysis [5, 23, 25]. Note that analysis performed on phase trajectories without prior transformations, or with square root transformations, gave qualitatively similar results.

Synchrony in epidemic amplitudes (or timing) between each pair of municipalities was measured as the correlation between each respective pair of ILI (or phase) trajectories. The average phase difference between each pair of phase trajectories was employed as a supplemental measure of synchrony in timing [23, 26]. Regional variation in synchrony was explored by mapping the correlation (or phase difference) between each municipality and a reference point, Oslo (the most densely populated municipality in Norway). To determine how synchrony varies as a function of the distance between municipalities, we estimated the spatial non-parametric correlation function (sncf) for the ILI and phase-angle time-series [27]. Specifically, we fit a smoothing spline curve using the ‘ncf’ library in R and generated 95% confidence intervals using 1000 bootstrap samples [28]. This analysis enabled estimation of the (potentially) nonlinear relationship underlying the distance between municipalities and the correlation in their epidemic trajectories.

Finally, to test the relationship between epidemic synchrony and a wider range of predictor variables, we also performed Mantel and partial Mantel tests with Spearman correlations [5, 28–30]. The former method tests for monotonic associations between a response and predictor variable that both vary across space, whereas the latter performs the same task whilst controlling for a third covariate. Our response variable was the pairwise correlation in ILI (or phase) trajectories between municipalities; and our predictor variables were the great-circle distance between those municipalities; the product of their population sizes; the absolute difference in their average altitude; the pairwise correlation in their temperature and specific humidity conditions; and the average number of airline passengers traveling between them. The choice of distance, population size, temperature, specific humidity, and airline travel (a proxy for population mobility) were motivated by previous influenza studies [5–7, 9–12, 21, 29]. Altitude was chosen as an additional covariate to assess whether mountainous regions in Norway provide natural barriers against influenza transmission (S1 Fig). This choice was motivated by previous work demonstrating the de-synchronizing effect of other geographical features, such as rivers and bays, on spatial dynamics of measles and rabies [31, 32]. Tests were performed with 10,000 permutations to determine the significance of each correlation coefficient [30]. For further details see S1 Text.

To explore patterns of transmission across national borders, the above analyses were repeated with additional data from Denmark and Sweden. Weekly time-series were aggregated by county from 2001–2014 in accordance with the common spatiotemporal resolution of the data. Of the resulting 45 time-series, eight failed our low sample size tests and were discarded from further analysis (see S5 Fig). The discarded series were exclusively from Sweden, and likely reflect lower sampling of laboratory data (particularly before the 2009/10 pandemic), rather than reduced influenza activity [33]. Of the remaining 37 time-series, the Swedish lab confirmed data were still substantially lower in magnitude than the ILI data from Denmark and Norway, and thus unsuitable for comparison of epidemic amplitudes. However, phase-angle trajectories should be robust to differences in sample size as the series are standardized and de-trended during the reconstruction procedure. We therefore limited our cross-border analyses to synchrony in epidemic timing using the corresponding phase-angle trajectories (reconstructed as described above). To test the robustness of the phase-angle reconstructions, we repeated our analyses with downsampled data from Norway and Denmark that were equivalent in magnitude to Sweden (see S1 Text for further details). We also compared the average epidemic center of mass as an alternative measure of synchrony in timing (S1 Text).

Finally, to compare our findings with those from a demographically and climatically diverse temperate region, the sncf analyses were repeated with the US ILI data and corresponding phase-angle trajectories. Two approaches were used to reconstruct the US phase-angles: 1) by using the original year-round ILI data; and 2) by first removing weeks 21–39 of each year to mimic the temporal distribution of sampling in Norway. Comparing these approaches enabled us to assess the impact of the unsampled weeks on our estimates of synchrony in Norway. To control for spatiotemporal resolution, the Norwegian data were aggregated by county from 2002–2009. To control for differences in the geographic scale of each country, we developed an algorithm to partition the contiguous US into an array of distinct transects of similar size and shape to Norway (see S1 Text for further details). The resulting 84 transects represented a range of demographic and climatic gradients.

## Results

### Within Norway

**Fig 2:**
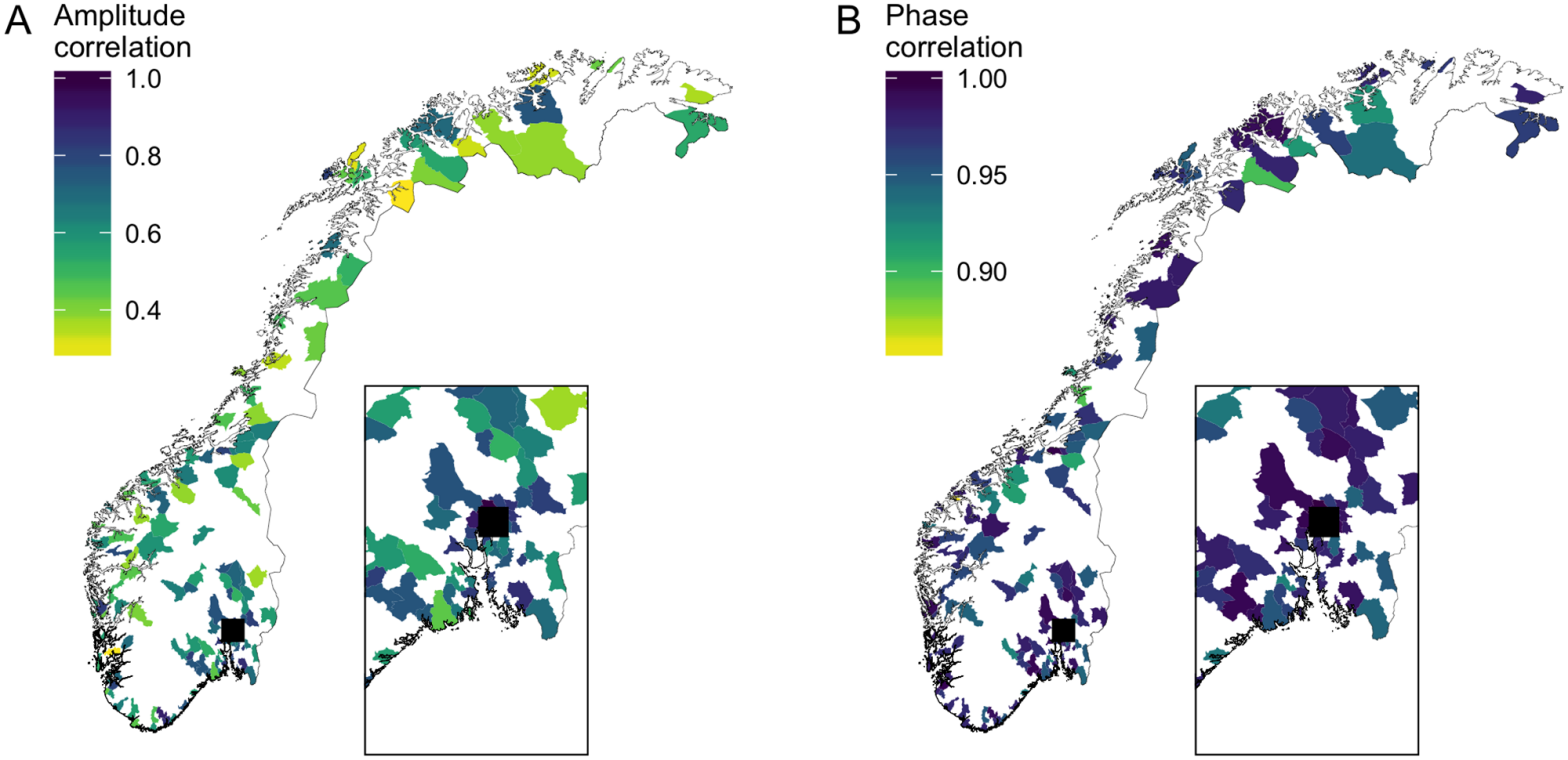
Geographic distribution of synchrony in epidemic amplitude and timing. Colors indicate the correlation in ILI (A) and phase-angle (B) trajectories between each municipality and Oslo; municipalities for which data were discarded or unavailable are shown in white. Oslo is indicated by the black square and the area surrounding the capital is enlarged in the inset box for clarity.

Mapping the pairwise correlations in ILI and phase-angle trajectories revealed that influenza epidemics are highly synchronized across Norwegian municipalities, particularly with respect to epidemic timing (Fig 2). Although some spatial structure is evident in the synchrony of epidemic amplitudes, with municipalities farther from Oslo tending to be less correlated (Fig 2A), the corresponding phase correlations and phase differences suggest epidemic timing is more evenly distributed (Fig 2B, S6 Fig). Most strikingly, we found strong phase correlations between Oslo and a number of distant northern municipalities.

**Fig 3:**
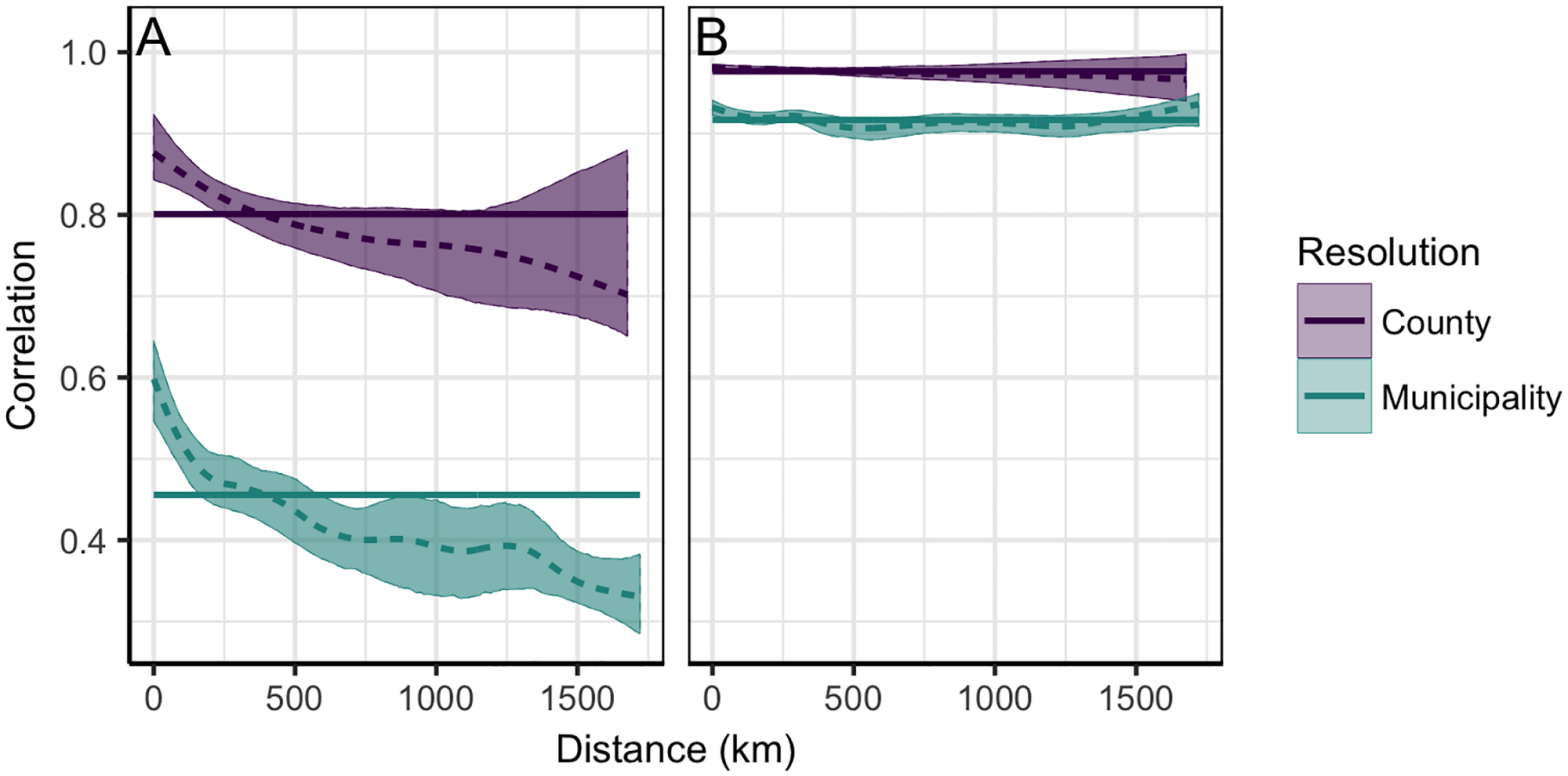
Relationship between synchrony and distance in Norway. Spatial non-parametric correlation function applied to Norwegian ILI (A) and phase-angle (B) trajectories. Colors indicate the spatial resolution i.e. municipality-level (blue) or county-level (purple). Solid lines represent the average synchrony across all regions; dashed lines depict the predicted relationship between synchrony and distance; and shaded regions are the 95% confidence intervals.

These findings were further confirmed by the sncf analysis. First, we estimated high levels of synchrony across the country, particularly in epidemic timing (Fig 3, solid blue lines). Moreover, as distance increased, the decline in synchrony was sharper for epidemic amplitudes than timing (Fig 3, dashed blue lines). This indicates a stronger impact of distance and thus a greater degree of spatial structuring. Finally, the unusual increase in phase correlations beyond 1200 kilometers (km) suggests close synchronicity in timing between geographically distant areas, as noted above for Oslo and certain northern municipalities.

Using Mantel tests we found that distance, population size, temperature, and specific humidity conditions are all moderately associated with synchrony in epidemic amplitudes and timing (*p* ≤ 0.002, Table 1). In contrast, average altitude was only weakly associated (*p* < 0.002, S1 Table), and there was no association with the average number of airline passengers traveling between municipalities (*p* = 0.42, S1 Table). After controlling for each significant predictor using partial Mantel tests, only population size remained moderately associated. In particular, specific humidity and temperature were not significant after controlling for distance, distance was not significant after controlling for temperature, and altitude was not significant after controlling for population size. This is likely due to correlations between specific humidity and distance (−0.98, *p* < 0.0002), distance and temperature (−0.96, *p* < 0.0001), and altitude and population size (−0.28, *p* < 0.0001). Moreover, we found that all municipalities are highly correlated with respect to their temperature and humidity time-series (across municipalities, temperature correlations ≥ 0.78, and humidity correlations ≥ 0.74). This suggests a substantial degree of spatial homogeneity in climatic conditions.

**Table 1:** Mantel tests at the municipality-level.

	Amplitude correlations		Phase correlations	
	Correlation	*p*-value	Correlation	*p*-value
*Mantel tests*				
Population*	0.40	0.0002	0.30	0.0002
Distance	−0.34	0.0001	−0.17	0.001
Humidity^†^	0.33	0.0002	0.16	0.002
Temperature^‡^	0.35	0.0002	0.17	0.002
*partial Mantel tests*				
Population, adjusted for:				
Distance	0.36	0.0002	0.27	0.0002
Humidity	0.37	0.0002	0.28	0.0002
Temperature	0.37	0.0002	0.28	0.0002
Distance, adjusted for:				
Population	−0.30	0.0001	−0.12	0.01
Humidity	−0.09	0.01	−0.07	0.046
Temperature	−0.03	0.21	−0.03	0.22
Humidity, adjusted for:				
Population	0.30	0.0002	0.12	0.009
Distance	−0.014	0.37	−0.04	0.18
Temperature	−0.08	0.06	0.05	0.19
Temperature, adjusted for:				
Population	0.32	0.0002	0.13	0.008
Distance	0.07	0.05	0.01	0.40
Humidity	0.13	0.005	0.07	0.10

Mantel and partial Mantel tests using Spearman correlations to detect associations between the amplitude and phase synchrony of Norwegian municipalities and a number of predictor variables.

* represents the product of population sizes for each municipality pair.

^†^ represents the correlation in specific humidity between each municipality pair.

^‡^ represents the correlation in temperature between each municipality pair.

### Across Norway, Sweden and Denmark

**Fig 4:**
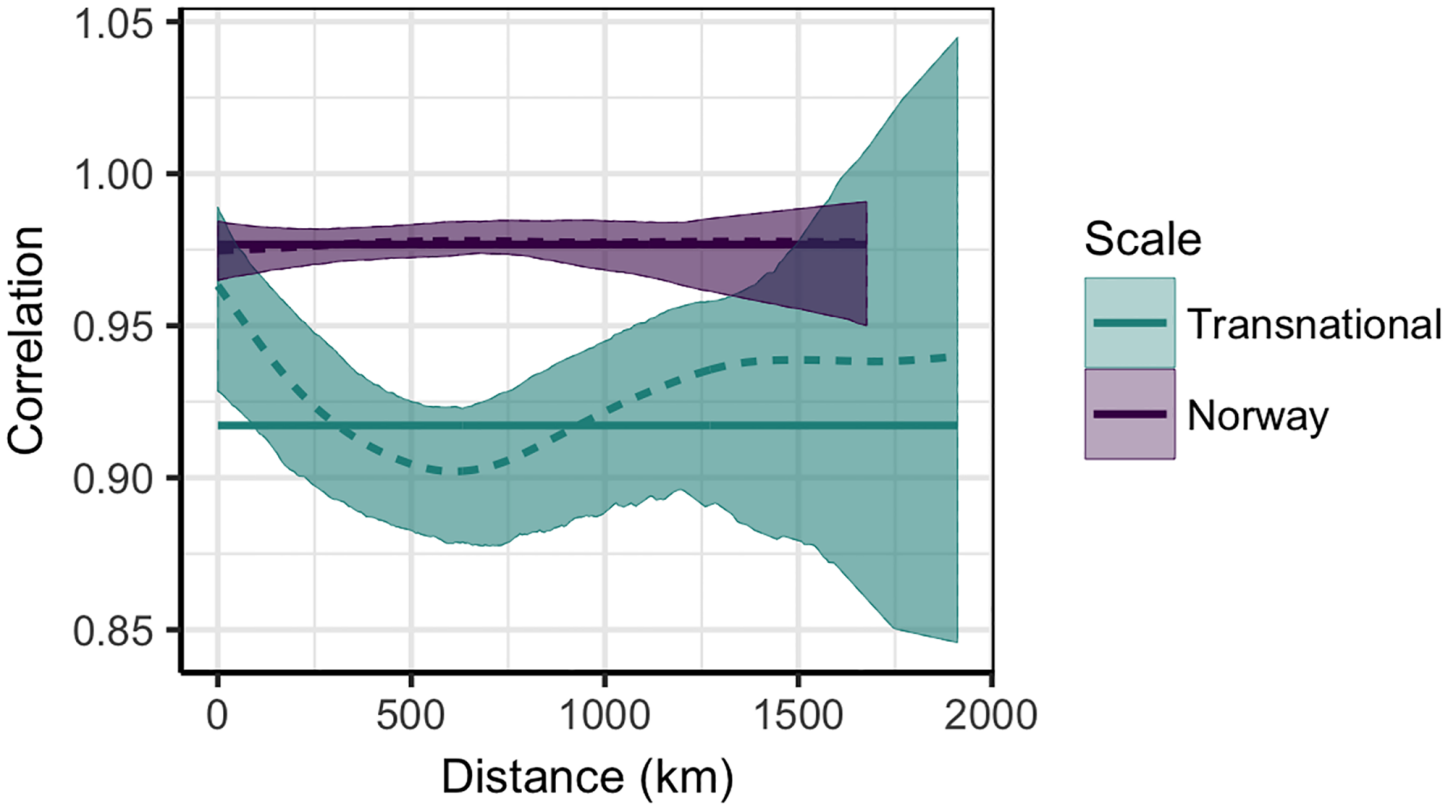
Relationship between synchrony and distance at a larger spatial scale. Spatial non-parametric correlation function for Norwegian, Swedish, and Danish counties (blue). The corresponding relationship amongst Norwegian counties is included for reference (purple). Solid lines represent the average synchrony across all regions, dashed lines depict the predicted relationship between synchrony and distance, and shaded regions are the 95% confidence intervals.

Extending the geographical scale to Sweden and Denmark required aggregating data by county rather than municipality. To test the impact of this change in resolution on our synchrony estimates, we first compared municipality and county-level sncf curves within Norway. Although synchrony increased for both phase-angle and ILI trajectories at the county-level, the former were substantially less affected (Fig 3) and are thus the more robust measure to compare across spatial resolutions. Subsequently incorporating Swedish and Danish phase-angles in the county sncf analysis revealed lower levels of synchrony, and a more pronounced relationship between synchrony and distance, than within Norway alone (Fig 4).

**Fig 5:**
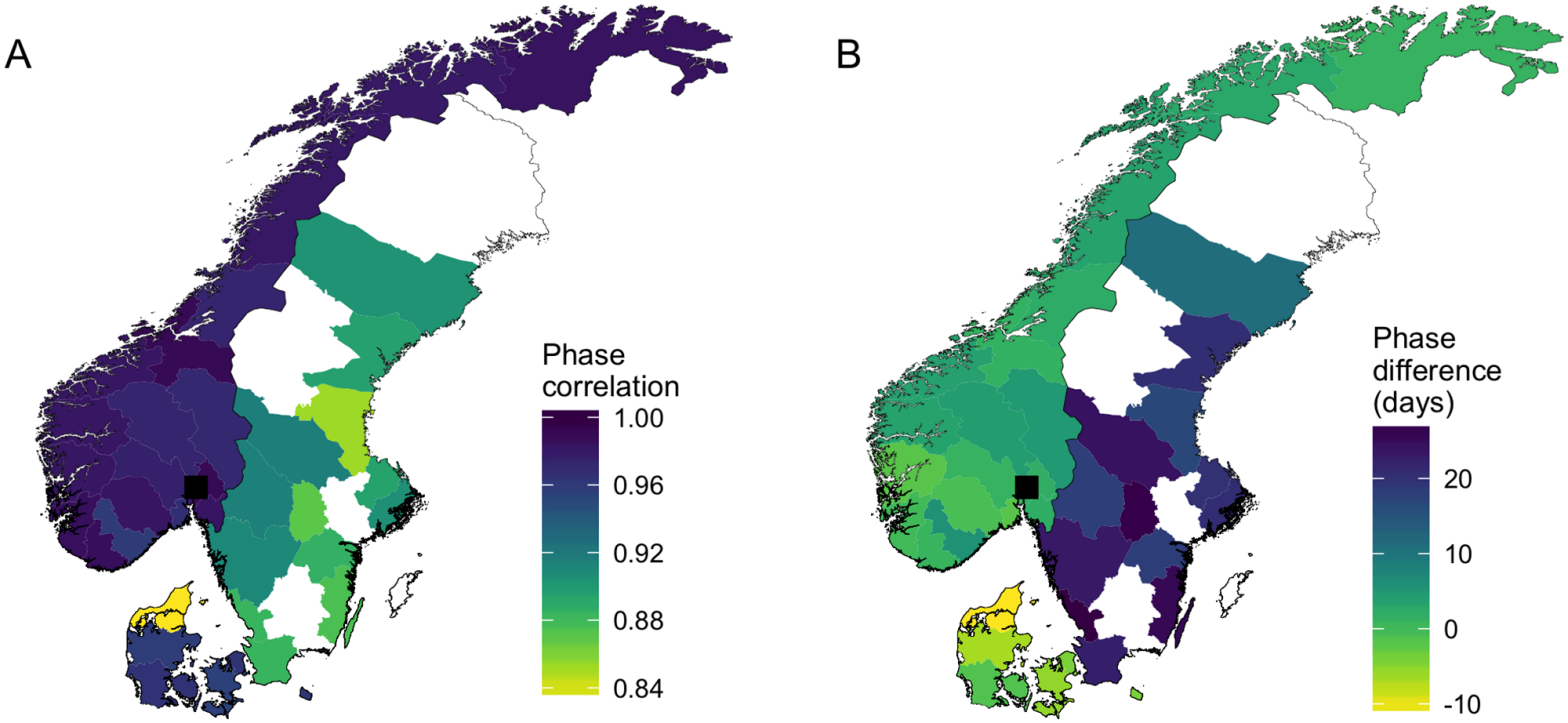
Geographic distribution of synchrony in epidemic timing between counties. Colors indicate the correlation in phase-angles (A) and the average phase difference (B) between each county and Oslo (marked by the black square); counties for which data were discarded are shown in white. A positive (negative) phase difference indicates epidemics tend to follow (precede) those in Oslo.

To explore the spatial structure of phase synchrony, we then mapped the correlation between each county and our reference point, Oslo. We found that epidemics in Denmark and Norway were more synchronized with each other than with those in Sweden (Fig 5A), and that this is likely driven by a delay in Swedish epidemics of up to three weeks (Fig 5B). These results held when center of mass was used as an alternative measure of epidemic timing (S7 Fig), and when the ILI data from Norway and Denmark were first downsampled to the magnitude of the Swedish data (S8 Fig).

Finally, Mantel tests were performed to detect potential drivers of synchrony across national borders. In addition to the distance, population size, temperature, and humidity predictors outlined above, we included an additional binary variable to indicate whether two counties share the same country (0) or not (1). This binary variable was the most strongly associated predictor, and was the only variable to remain significant whilst controlling for all other predictors (*p* ≤ 0.0001, S2 Table). Using the downsampled data from Norway and Denmark did not change these conclusions (S3 Table), nor did excluding Swedish counties from the analysis (S4 Table).

### Comparison with the United States

**Fig 6:**
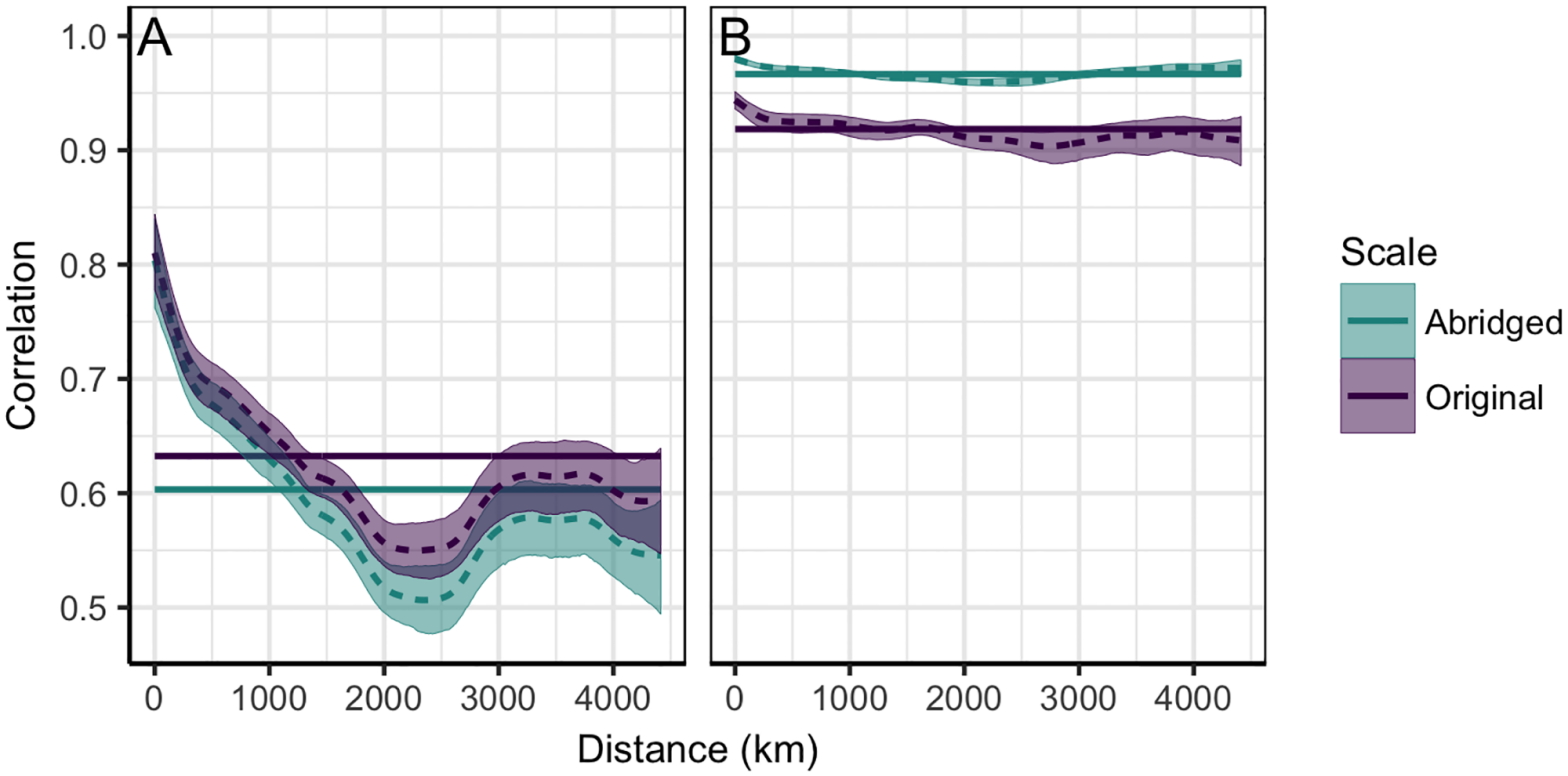
Impact of sampling on the relationship between synchrony and distance. Spatial non-parametric correlation function applied to US ILI (A) and phase-angle (B) trajectories. Colors indicate the temporal sampling distribution i.e. original year-round (purple) or abridged with weeks 21–39 removed (blue). Solid lines represent the average synchrony across all regions, dashed lines depict the predicted relationship between synchrony and distance, and shaded regions are the 95% confidence intervals.

One notable difference between the US and Norwegian reporting of seasonal influenza is that the former remains consistent year-round, whereas the latter is suspended each year during weeks 21–39. We took advantage of the consistent temporal sampling in the US to investigate the impact of missing weeks on our Norwegian synchrony estimates. Specifically, for synchrony in epidemic amplitudes, we compared results using the original US ILI time-series to those obtained from an abridged dataset in which weeks 21–39 were removed. These abridged data were less synchronized than the original series, suggesting that missing weeks lead to an underestimation of amplitude synchrony (Fig 6A). For synchrony in epidemic timing, we compared results using phase angles reconstructed from the original time-series to those reconstructed from the abridged data. In contrast to the effect on epidemic amplitudes, the abridged phase-angles were more synchronized than the original trajectories, suggesting an overestimation of synchrony in epidemic timing (Fig 6B).

#### US transects

To compare synchrony in Norway to that in the US, we first controlled for differences in temporal sampling by using the abridged US data. To control for geographic scale, we then partitioned the US into 84 transects of similar size and shape to Norway (S9 and S10 Figs). Compared to Norwegian counties, the majority of these transects had greater mean and variance in temperature, humidity, and population sizes (S11 Fig). Estimates of synchrony for each transect, and for Norway, were obtained from the respective sncf (see for example S12 Fig). For all transects, both amplitude and phase synchrony were lower than that of Norway (S13 Fig).

## Discussion

Guided by previous work, we expected to find a strong spatial signature in Norwegian influenza epidemics. In particular, we hypothesized that epidemics would originate in larger, well-connected cities and later diffuse into more isolated regions, and that climatic conditions would modulate the speed of this spread [5, 6]. Instead, we found surprisingly little spatial variation in the synchrony of epidemics, with only population size moderately mediating this synchrony. Expanding the geographic scale of our analysis to include Sweden and Denmark did not substantially change these results. In addition, we hypothesized that spatial synchrony in Norway would be lower than US transects of comparable size, given the smaller population of the former country (approximately 4.9 million, compared to a mean transect population size of 44.9 million (range: 3.8 million–178.6 million)). Unexpectedly, however, we found the opposite. Overall, our results provide novel insights into the spatiotemporal spread of influenza in Norway, Sweden, and Denmark. Moreover, the unusually high degrees of synchrony, despite relatively small population sizes, highlight the importance of studying diverse environmental and demographic regions if influenza dynamics are to be better understood.

### Within Norway

Within Norway we found greater synchrony in epidemic timing than epidemic amplitudes. This echoes previous studies of influenza in the US and measles in the UK [5, 21, 23], and is likely driven by increased sensitivity of epidemic amplitudes to demographic noise in small populations. However, our predictions of phase synchrony are very high relative to these previous studies. Further investigation with the US data suggests this may be partly due to a lack of sampling during the summer (weeks 21–39). Caution must therefore be exercised when interpreting such incomplete data.

Also in agreement with previous literature, we find that distance has a stronger impact on synchrony in epidemic amplitudes than epidemic timing [5, 21]. This is unlikely to be driven by demographic noise as spatial signals should be obscured by low case counts rather than amplified. Other unlikely factors are temperature and humidity conditions: these were highly correlated with geographic distance and would thus be expected to modulate epidemic amplitudes and timing in similar ways. More plausible factors, that could differentially mediate the severity of epidemics across space, include local differences in influenza strain dynamics, prior immunity, and healthcare seeking behavior. These were outside the scope of this study, but would be interesting covariates to include in future work.

Specific humidity and temperature are parsimonious predictors of influenza virus transmission and survival and have been shown to modulate the timing and spatial spread of influenza epidemics in the US [6, 8, 10–13]. However, these variables were not significant in our analysis once we accounted for distance. Instead, the high correlations in humidity and temperature conditions across municipalities suggest country-wide similarities in climate that may promote synchrony across space. Although other climatic variables, such as sunlight levels, may also modulate influenza activity, these were not included in the present study as the underlying mechanisms are less well defined [34]. Future work could consider these as additional covariates.

Alternative drivers of synchrony that we were unable to address include high levels of population movements and external importations of influenza. In particular, the well-mixed pattern of timing could be explained by multiple distinct introductions from other countries. Although we did not identify air travel as a significant predictor of spatial synchrony, our data did not include international flights and may thus miss external seeding events. Furthermore, other modes of ground transportation are likely to be important for more localized movement between municipalities. Therefore, additional mobility data, such as age-stratified commuting patterns, are needed to understand how smaller scale movements influence transmission in this region, particularly for key age groups [5, 21, 29, 35–37].

More generally, the high levels of synchrony found in this relatively small population points to the open question of how much host mixing is needed to drive strong spatial synchrony in influenza transmission. Dynamics in small populations are often challenging to analyze due to demographic noise obscuring epidemic signals. However, the clear impact of distance on amplitude synchrony (Fig 3) indicates there is a strong signal in these data despite this fact. Small populations can therefore provide useful insight into the local dynamics of influenza, and more quantitative work in this area is needed to elucidate how host mixing, climate, and demography impact the spread of seasonal epidemics.

### Across Norway, Sweden and Denmark

Aggregating data across national reporting systems is a vital step towards characterizing the international spread of influenza. However, care must be taken when reconciling data from different sources and spatiotemporal resolutions. Before extending our analysis beyond Norway, we therefore compared estimates of synchrony at different resolutions, and found an increase at the county-level that was greater for epidemic amplitudes than timing (Fig 3). Similar patterns have been found in the US when comparing city and state-level data [21], and may be explained by considering the decrease in demographic noise at courser spatial resolutions. For example, when reconstructing phase-angles from the annual period of the ILI time-series, we remove noise at other frequencies. This may buffer the resulting phase trajectories against transient ILI fluctuations in small populations, and thus reduce their sensitivity to demographic noise. Overall, our findings suggest phase analysis may provide a more robust measure of spatial synchrony across different resolutions.

Incorporating data from Denmark and Sweden highlighted a striking systematic delay in the timing of epidemics in the latter country. Although the Swedish data represent confirmed influenza cases rather than ILI reports, previous work has shown these different sources consistently estimate the timing of influenza epidemics within a week of one another [17]. Our finding that Swedish epidemics are delayed by up to three weeks is outside this period of potential reporting bias. In addition, the robustness of phase trajectories to sample size, and the fact that downsampled Norwegian and Danish data produced equivalent results, suggest this lag represents a true phenomenon. Furthermore, these findings are consistent with previous reports of an eastward spread of influenza across Europe [16, 17, 38]. In particular, Alonso et al. (2015) found Swedish epidemics occurred, on average, two weeks after those in Denmark and Norway. This study compared laboratory confirmed cases of influenza across all three countries and thus does not suffer from inconsistent data sources.

The strong association between synchrony and the binary country variable, even after excluding the Swedish data, also suggests that country-specific factors favor greater synchrony within nations than between them. For instance, differences in school winter term times, such as a delay in the reconvening of Swedish schools, may mediate mixing between school-age children and impact local patterns of spread via mechanisms we cannot investigate with the current data. Other potential candidates include inter-country variation in the promptness of case reporting, population movements, and healthcare seeking behavior [39].

### Comparison with the United States

We first took advantage of consistent temporal sampling in the US to investigate the impact of missing weeks on our Norwegian estimates of synchrony. We found that phase synchrony may be overestimated whereas amplitude synchrony may be underestimated, and propose that both cases can be explained by considering the respective timing of greatest spatial variation. For example, in the case of ILI trajectories, variation in amplitudes is greatest during the peak of the epidemics (i.e. during winter), whereas in summer amplitudes are more similar (i.e. all very small). Removing the summer weeks then removes the most correlated part of the time-series and thus causes synchrony to decrease. In contrast, variation in phase-angles should be greatest before epidemics begin to take off, when the underlying periodic signal is most obscured (i.e. near the transition between summer and winter). Removing this period would then increase correlation, and thus estimates of synchrony.

#### US transects

Partitioning the abridged US data into transects of similar size to Norway allowed us to compare synchrony between these countries whilst controlling for differences in sampling and geographic scale. We expected larger population sizes in the US transects to have a stronger synchronizing effect on influenza epidemics. Instead, we found that amplitude and phase synchrony were indiscriminately higher in Norway. One reason for this may be the lower mean and variance of Norwegian climatic conditions (S11 Fig). For example, lower mean humidity and temperature may facilitate increased survival and transmission of the influenza virus [11, 12], whilst lower variation in these conditions may promote tighter spatial coupling that overcomes the expected lag between small populations. However, we note caution when interpreting these results as there may be other important differences between Norway and the US transects that we were unable to explore. For example, we did not assess differences in vaccine uptake, healthcare seeking behavior, population movements, age distributions, or school term times. In particular, the spatial spread of the 2009/10 US pandemic was mediated by variation in school opening times, whereas population movements can parsimoniously explain the corresponding patterns of seasonal outbreaks [5, 8]. Further analyses could therefore include movement data, and differences in the duration and timing of school terms, as additional covariates.

Another caveat to our transect analysis is that the partitioning algorithm likely only captures a subset of all possible US transects. In addition, the coarse state-level resolution of the algorithm may have facilitated disproportionate representation of expansive and well-connected states in the central and southern regions. Therefore, although the transects we identified collectively spanned the entire country and included a range of population sizes and climatic conditions, we cannot guarantee that the resulting synchrony distribution is fully representative. Future work could explore this problem further by comparing both countries at finer spatial resolutions. This would allow more flexible partitioning of transect boundaries and greater sampling of irregular coastal and northern regions.

### Caveats

Additional caveats to note concern the type of data used in this study. Firstly, as the majority of our data are GP diagnosed cases of ILI, they may also include misclassified cases of other wintertime respiratory diseases, such as respiratory syncytial virus. However, sentinel ILI data are a robust indictor of influenza activity in Europe [17], and if misreporting is approximately uniform across space, it should not impact our general conclusions regarding spatial synchrony. Secondly, there were inconsistencies in our international data, as the Swedish time-series were confirmed influenza cases rather than ILI reports. As discussed in the previous sections, we believe our general conclusions are robust to this fact. However, we do note that eight counties in Sweden had to be discarded due to low sampling of confirmed cases. Although the remaining twelve are geographically, demographically, and climatically representative (see for example Fig 5 and S2 Fig), incorporating the discarded counties would provide a more complete analysis of spatial synchrony. Increased laboratory sampling in Sweden following the 2009/10 pandemic should facilitate such work in the future.

Thirdly, our analyses do not differentiate influenza cases by strain or subtype and thus cannot account for patterns of spatial spread that may be caused by concurrent circulation of multiple strains [40, 41]. For instance, well-mixed dynamics of aggregated influenza data could mask underlying structure in the local spread of distinct strains. The extent to which strain coexistence impacts overall spatial synchrony across small populations is an interesting area for future research. Finally, there may be regional and temporal differences in reporting rates that our analyses do not include, for example due to inaccessibility of local GP offices or increased reporting during the 2009/10 pandemic [42]. However, our general conclusions should be robust to such fluctuations as: 1) the sentinel system is designed to maintain even population representation and reporting frequencies across space [18, 19]; and 2) repeating analyses without the 2009/10 pandemic season did not significantly change our results (see for example S14 Fig).

### Concluding remarks

Using rich data from Norway, Sweden, and Denmark we characterized the spatial spread of influenza at different spatial resolutions and geographic scales. Although we found high degrees of epidemic synchrony, further comparison with the US suggested these estimates may be partly confounded by discontinuous temporal sampling. National surveillance systems are diverse with respect to sampling procedures, and our results highlight the challenges in analyzing influenza spread across regions where such differences are present. Nevertheless, important insights can be gained from careful studies that take sampling practices into account. In particular, future mechanistic models could be used to compare different sampling procedures and test their impact on the above analyses. Finally, incorporating data from the US enabled the comparison of influenza spread in two very different regions of the Northern Hemisphere. Contrary to expectations, US epidemics were less synchronized than those in Norway, demonstrating that further study of diverse environmental and demographic settings is needed to better understand the key mechanisms underlying influenza spread.

## Supporting information

S1 Text Additional data and analysis. Details of additional covariate data and statistical methods referenced in the main text.
(PDF)

S1 Table Mantel tests at the municipality-level. Mantel and partial Mantel tests using Spearman correlations to detect associations between the amplitude and phase synchrony of Norwegian municipalities and a number of additional predictor variables.
(PDF)

S2 Table Mantel tests at the county-level. Mantel and partial Mantel tests using Spearman correlations to detect associations between the phase synchrony of Norwegian, Swedish, and Danish counties and a number of predictor variables.
(PDF)

S3 Table Mantel tests at the county-level using downsampled data. Mantel and partial Mantel tests using Spearman correlations to detect associations between the phase synchrony of Norwegian, Swedish, and Danish counties and a number of predictor variables (using downsampled Norwegian and Danish data).
(PDF)

S4 Table Mantel tests at the county-level, excluding Sweden. Mantel and partial Mantel tests using Spearman correlations to detect associations between the phase synchrony of Norwegian and Danish counties and a number of predictor variables (after excluding the Swedish data).
(PDF)

S1 Fig Demographic and environmental covariates within Norway. Municipality-level measures of population size (A), specific humidity (B), temperature (C), altitude (D), and airline travel (E). For details on how these data were obtained, see S1 Text.
(PDF)

S2 Fig Demographic and environmental covariates across Norway, Sweden and Denmark. County-level measures of population size (A), vapor pressure (B), and temperature (C). For details on how these data were obtained, see S1 Text.
(PDF)

S3 Fig Discarded municipality-level data. Norwegian ILI time-series that were removed from our analysis due to failure of a Box-Pierce white noise test or the presence of at least two seasons with less than ten cases reported.
(PDF)

S4 Fig Norwegian municipality-level data. Norwegian ILI time-series that were used in all municipality-level analyses. Each row represents a distinct municipality, and these are ordered from top to bottom by decreasing latitude. Blank regions represent weeks for which we do not have data.
(PDF)

S5 Fig County-level data. Norwegian, Swedish, and Danish county time-series. Red trajectories indicate that the data were removed from further analysis due to failure of a Box-Pierce white noise test or the presence of at least two seasons with less than ten cases reported; series that were kept for further analysis are depicted with blue trajectories. The background color of each plot indicates the country in which each county is located.
(PDF)

S6 Fig Phase differences at the municipality-level. Average phase differences between each Norwegian municipality and Oslo. Oslo is indicated by the black square and the area surrounding the capital is enlarged in the inset box for clarity. A positive (negative) phase difference indicates epidemics tend to follow (precede) those in Oslo.
(PDF)

S7 Fig Average epidemic center of mass. Colors indicate the average center of mass of epidemics (in calendar weeks) for each Norwegian, Swedish, and Danish county; counties for which data were discarded are shown in white. Oslo is indicated by the black square.
(PDF)

S8 Fig Synchrony in epidemic timing using downsampled data from Norway and Denmark. Colors indicate the correlation in phase-angles (A) and the average phase difference (B) between each county and Oslo (marked by the black square); counties for which data were discarded are shown in white.
(PDF)

S9 Fig All US transects. States in blue were those included in each transect.
(PDF)

S10 Fig Transect distribution. Each state is colored according to the proportion of transects in which it is included.
(PDF)

S11 Fig Transect variation in population and climate. Mean and standard deviation of city humidity conditions (measured as vapor pressure) (A), population size (B), and temperature (C) within each US transect; red points indicate the corresponding values across Norwegian counties.
(PDF)

S12 Fig Example US transect. Top: blue states on the map were those included in the transect. Bottom: Spatial non-parametric correlation function comparing synchrony between the US transect (blue) and Norway (purple) for (A) ILI trajectories and (B) phase-angle trajectories. Solid lines represent the average synchrony across all regions, dashed lines depict the predicted relationship between synchrony and distance, and shaded regions are the 95% confidence intervals.
(PDF)

S13 Fig Synchrony of all US transects. Average synchrony estimates for 84 US transects obtained by applying the spatial non-parametric correlation function to the abridged data of (A) ILI trajectories and (B) phase-angle trajectories. Points mark transect centroids and colors represent the corresponding synchrony estimate. Inset panels show the distribution of transect synchrony estimates; the estimate for Norway is marked by the dashed line.
(PDF)

S14 Fig Relationship between synchrony and distance in Norway, with and without the 2009/10 pandemic. Spatial non-parametric correlation function for municipalities in Norway with (purple) and without (blue) the inclusion of the 2009/10 pandemic for (A) ILI trajectories and (B) phase-angle trajectories. Solid lines represent the average synchrony across all regions; dashed lines depict the predicted relationship between synchrony and distance; and shaded regions are the 95% confidence intervals.
(PDF)
