# Supplementary material for: Analysis of multi-level spatial data reveals strong synchrony in seasonal influenza epidemics across Norway, Sweden, and Denmark
Source: PLoS One. 2018 May 17;13(5):e0197519. doi: 10.1371/journal.pone.0197519 (PMC5957349; doi:10.1371/journal.pone.0197519)
Supplement: S1 Text — Details of additional covariate data and statistical methods referenced in the main text. (PDF) [file pone.0197519.s001.pdf]

Supplementary Text

Analysis of multi-level spatial data reveals strong synchrony in seasonal influenza epidemics across Norway, Sweden, and Denmark

1 Demographic, geographic, and climatic data

Population estimates Norwegian population estimates were obtained from Statistics Norway (http://www.ssb.no/en, accessed 12 March 2015) at the municipality level (one point estimate from 2014) and the county level (annual estimates from 2006–2014). One estimate for each county was obtained by taking the median value of each respective time-series. Annual population estimates for Sweden from 2006–2014 were obtained at the county level from Statistics Sweden (http://www.scb.se/en, accessed 26 April 2016), and quarterly Danish population estimates from 2008–2014 were obtained at the regional level from Statistics Denmark (http://www.statistikbanken.dk, accessed 26 Apr 2016). One estimate for each county (or region) was obtained by taking the median value of each time-series. US population estimates were obtained from the 2000 US census (http://www.census.gov/popest/, accessed 1 January 2014). Note that the temporal range of the population data were limited to information that was publicly available at the time of analysis. Wherever direct comparisons were made (e.g. across counties in Sweden, Norway, and Denmark), every effort was made to keep the data as consistent as possible.

Geographic data For all countries, latitude and longitude coordinates were assigned as the geographic centroid of each municipality (or county), and the great-circle distance between each pair of respective regions was calculated using the R package 'geosphere' [1]. Altitudinal data for each Norwegian municipality was obtained from Statistics Norway (accessed 10 March 2016) and listed the total area (in km^2) contained within each of a series of altitude bands (0–59m, 60–159m, ..., 2100–2700m above sea level). For each municipality, the average altitude was calculated as the sum of the midpoints of each altitude band weighted by the proportion of the total municipal area contained within that band.

Movement data Data on the quarterly numbers of airline passengers traveling between all commercial airports in Norway from 2009–2014 were obtained from Statistics Norway (accessed 15 April 2015). Airports were matched to the

corresponding municipality in which they were located using airport names provided in the data and publicly available information on airport geographic locations (https://www.flightpedia.org/airports-in-norway.html, accessed 15 April 2015). For each municipality pair, the amount of air passenger flow was quantified as the mean number of passengers traveling between their respective airports across all quarterly observations in the data. Note that this number was zero if there was no airport located in one, or both, of the municipalities. These data are therefore limited as they do not quantify pairwise movements between all municipalities in our study.

Climatic data Norwegian climatic data were obtained from the Japanese 55-year Reanalysis (JRA-55) dataset provided by the Research Data Archive (RDA) at the National Center for Atmospheric Research (NCAR) (http://rda.ucar.edu, accessed 6 October 2015 and 9 August 2017). Specific humidity (kg/kg) and temperature (°C) values at 2m above sea level were obtained every six hours from 1998–2014 at a grid cell resolution of 0.562° x 0.562°. To obtain daily values we calculated the mean specific humidity and temperature for each day and grid cell.

To obtain time-series for each municipality (or county) (and thus match the spatial resolution of the epidemiological data) we compared two different approaches to calculating daily humidity values:

1. we took each municipality (or county), i, in turn and calculated the mean daily specific humidity value across all grid cells with at least 10% of their total area contained within i, similar to previous methods [2].

2. we took the daily specific humidity value of the grid cell closest to the geographic centroid of i.

Since the time series obtained from both methods were highly correlated across regions (estimate = 0.986, p-value = 0.0002) we report humidity and temperature results for the latter (centroid) method only.

We also calculated the daily specific humidity anomalies for each municipality, defined for each day, d, as the deviation from the average specific humidity on d across all years in the dataset (e.g. 1998–2014). The resulting data were highly correlated with the original specific humidity data (estimate = 0.995, p-value = 0.0002), and so we report results for the original data only.

To validate our results against alternative data, we also obtained monthly vapor pressure (hPa) measurements at 0.5° grid cell resolution from the TS version 3.23 global dataset provided by the Climatic Research Unit (CRU) at the University of East Anglia (https://crudata.uea.ac.uk/cru/data/hrg/, accessed 25 October 2016) [3]. Spatial resolution was matched to the epidemiological data using the centroid method described above, and the resulting time series were highly correlated with the Norwegian daily specific humidity dataset. Specifically, after smoothing the specific humidity data to monthly intervals, the correlation between the datasets was 0.96; and after transforming both raw datasets to similarity matrices and performing mantel tests, the correlation was 0.86, with a p-value of 0.0002.

To compare climatic conditions in Norway, Sweden, and Denmark, whilst minimizing computational intensity, we used

the monthly CRU vapor pressure data from 2001–2014. We also obtained monthly temperature records for 2001–2014 from the same data source (accessed 9 August 2017). Spatial resolution for both climatic datasets was matched to the county-level epidemiological data using the centroid method. Finally, to compare climatic conditions in Norway and the US we used the monthly CRU vapor pressure and temperature measurements from 2002–2009. Again, the centroid method was used to aggregate the resulting Norwegian data by county and US data by city.

2 Mantel tests

To compare the influence of possible predictor variables on epidemic synchrony, Mantel and partial Mantel tests were performed with the ILI and phase-angle trajectories [4–6]. These methods test for associations between the synchrony of municipalities and their 'similarity' in terms of demographic or environmental covariates (e.g. the geographic distance between them or their respective population sizes). In particular, partial Mantel tests are used to explore the relationship between synchrony and a particular predictor whilst controlling for another predictor.

First, symmetric matrices of synchrony in epidemic amplitude (timing) were computed such that each element, $m_{i,j}$, represents the correlation in ILI (phase) trajectories between municipalities i and j. Similar symmetric matrices were then computed for each predictor variable with elements $\{i, j\}$ as follows: a distance matrix representing the great-circle distance between municipalities i and j; a population matrix with the product of the population sizes of i and j; an altitude matrix with the absolute difference in average altitude between i and j; a humidity matrix with the correlation in specific humidity conditions between i and j; a temperature matrix with the correlation in temperature conditions between i and j; and an airline matrix with the average number of airline passengers traveling between i and j. Mantel and partial Mantel tests were then conducted with the 'ncf' library in R, using 10,000 permutations to determine the significance of each correlation coefficient [6, 7].

3 Downsampling ILI data

The data from Norway and Denmark consist of weekly numbers of ILI cases, whereas the Swedish data represent weekly numbers of positive confirmed cases of influenza. As a result, the Swedish data are lower in magnitude to that of Norway and Denmark. To explore the impact of this discrepancy on our main results, we repeated the same analyses with downsampled data from Norway and Denmark, that was calculated as follows. In each season, the total number of Swedish cases is, on average, 10% of the total number of cases in Norway or Denmark. We therefore downsample the Norwegian and Danish data to approximately 10% by drawing the new number of cases week w, I_w, from a Binomial (n_w, p) distribution, where n_w is the original number of cases reported that week and $p = 0.1$ is the probability of observing a

case in the new downsampled data. This leads to an expected number of cases in each week that is 10% of the original number of cases, and thus closer in magnitude to the Swedish data.

4 Average epidemic center of mass

For a particular region, the timing of each influenza epidemic can be captured by calculating its center of mass. Starting from the first week of sampling in each season, the center of mass, or mean timing, of the epidemic is calculated as

$$\frac{\sum_w w * I_w}{\sum_w I_w},$$

where $w \in [-26, 26]$ is an index for each week of the year, and I_w is the incidence reported in week w. Note that w is ordered so that w = (1, 2, ..., 26) corresponds to calendar weeks (1, 2, ..., 26) and w = (-26, -25, ..., -1) corresponds to calendar weeks (27, 28, ..., 52). Thus each season running from calendar week 40 of one year to calendar week 20 of the following year is indexed by weeks (-13, -12, ..., -1, 1, 2, ..., 20). To obtain a single value for each region, we calculated the average center of mass across all seasons. A positive value indicates epidemics occur, on average, after January 1st, whereas a negative value indicates they tend to occur before.

5 US transects

The shape of Norway can be characterized by its strong linear structure: the mainland spans approximately 1750 kilometers (km) from north to south, in comparison to approximately 400 km across its widest point. To control for the difference in geographic scale between the US and Norway, we developed an algorithm to partition the US into transects that captured this linear structure. The algorithm proceeds as follows.

1. For each contiguous US state, find its centroid.
2. From each state centroid, project straight lines, 1750 km in length, out at eight equally spaced angles (0, 45, 90, 135, 180, 225, 270, and 315°).
3. Discard line segments for which the end point falls outside the contiguous US border.
4. For each remaining line segment, find all the states it passes through (including the states at each end point). Each group of states then forms one transect.
5. Manually check the resulting transects for general shape.

This algorithm resulted in 84 transects similar in size to Norway (S9 Fig).

References

1. Hijmans RJ, Williams E, Vennes C, Hijmans MRJ. Package 'geosphere'. The Comprehensive R Archive Network. 2015;Available from: https://cran.r-project.org/web/packages/geosphere/geosphere.pdf.
2. Shaman J, Pitzer VE, Viboud C, Grenfell BT, Lipsitch M. Absolute humidity and the seasonal onset of influenza in the continental United States. PLoS Biology. 2010;8(2):e1000316.
3. Harris I, Jones P, Osborn T, Lister D. Updated high-resolution grids of monthly climatic observations–the CRU TS3. 10 Dataset. International Journal of Climatology. 2014;34(3):623–642.
4. Viboud C, Bjørnstad ON, Smith DL, Simonsen L, Miller MA, Grenfell BT. Synchrony, waves, and spatial hierarchies in the spread of influenza. Science. 2006;312(5772):447–451.
5. Stark JH, Cummings DA, Ermentrout B, Ostroff S, Sharma R, Stebbins S, et al. Local variations in spatial synchrony of influenza epidemics. PloS one. 2012;7(8):e43528.
6. Legendre P, Legendre L. Numerical ecology: Second Edition. Elsevier: Amsterdam, the Netherlands; 1998.
7. Bjørnstad ON. Package 'ncf'. The Comprehensive R Archive Network. 2009;p. 1–38. Available from: http://cran.r-project.org/web/packages/ncf/ncf.pdf.
