# Supplementary material for: Analysis of multi-level spatial data reveals strong synchrony in seasonal influenza epidemics across Norway, Sweden, and Denmark
Source: PLoS One. 2018 May 17;13(5):e0197519. doi: 10.1371/journal.pone.0197519 (PMC5957349; doi:10.1371/journal.pone.0197519)
Supplement: S1 Table — Mantel and partial Mantel tests using Spearman correlations to detect associations between the amplitude and phase synchrony of Norwegian municipalities and a number of additional predictor variables. (PDF) [file pone.0197519.s002.pdf]

Table S1. Mantel tests at the municipality-level.

	Amplitude correlations		Phase correlations	
	Correlation	p-value	Correlation	p-value
Mantel tests				
Altitude[‡]	−0.15	0.001	−0.14	0.003
Air travel[II]	−0.006	0.42	0.008	0.39
partial Mantel tests				
Population, adjusted for altitude	0.37	0.0002	0.27	0.0003
Distance, adjusted for altitude	−0.34	0.0001	−0.16	0.001
Humidity, adjusted for altitude	0.33	0.0002	0.15	0.002
Temperature, adjusted for altitude	0.35	0.0002	0.16	0.002
Altitude, adjusted for:				
Population	−0.04	0.16	−0.07	0.07
Distance	−0.14	0.003	−0.13	0.006
Humidity	−0.14	0.001	−0.14	0.004
Temperature	−0.14	0.002	−0.14	0.005

Mantel and partial Mantel tests using Spearman correlations to detect associations between the amplitude and phase synchrony of Norwegian municipalities and a number of additional predictor variables.

‡ represents the absolute difference in average altitude between each municipality pair.

II represents the average number of airline passengers traveling between each municipality pair.
