# Supplementary material for: Analysis of multi-level spatial data reveals strong synchrony in seasonal influenza epidemics across Norway, Sweden, and Denmark
Source: PLoS One. 2018 May 17;13(5):e0197519. doi: 10.1371/journal.pone.0197519 (PMC5957349; doi:10.1371/journal.pone.0197519)
Supplement: S2 Table — Mantel and partial Mantel tests using Spearman correlations to detect associations between the phase synchrony of Norwegian, Swedish, and Danish counties and a number of predictor variables. (PDF) [file pone.0197519.s003.pdf]

Table S2. Mantel tests at the county-level.

	**Phase correlations**	
	Correlation	p-value
Mantel tests		
Population*	−0.21	0.046
Distance	−0.11	0.12
Humidity	0.13	0.10
Temperature	−0.04	0.38
Region†	−0.68	0.0001
partial Mantel tests		
Population, adjusted for:		
Distance	−0.23	0.04
Humidity	−0.22	0.04
Temperature	−0.21	0.05
Region	−0.04	0.36
Distance, adjusted for:		
Population	−0.14	0.08
Humidity	−0.001	0.47
Temperature	−0.28	0.0005
Region	0.12	0.09
Humidity, adjusted for:		
Population	0.14	0.09
Distance	0.06	0.25
Temperature	0.24	0.007
Region	−0.12	0.10
Temperature, adjusted for:		
Population	−0.02	0.43
Distance	−0.26	0.0005
Humidity	−0.20	0.009
Region	−0.21	0.009
Region, adjusted for:		
Population	−0.66	0.0001
Distance	−0.68	0.0001
Humidity	−0.68	0.0001
Temperature	−0.69	0.0001

Mantel and partial Mantel tests using Spearman correlations to detect associations between the phase synchrony of Norwegian, Swedish, and Danish counties and a number of predictor variables.

* represents the product of population sizes for each municipality pair.
† binary variable indicating whether two counties are in the same country (0) or not (1).
