# Supplementary material for: Analysis of multi-level spatial data reveals strong synchrony in seasonal influenza epidemics across Norway, Sweden, and Denmark
Source: PLoS One. 2018 May 17;13(5):e0197519. doi: 10.1371/journal.pone.0197519 (PMC5957349; doi:10.1371/journal.pone.0197519)
Supplement: S3 Table — Mantel and partial Mantel tests using Spearman correlations to detect associations between the phase synchrony of Norwegian, Swedish, and Danish counties and a number of predictor variables (using downsampled Norwegian and Danish data). (PDF) [file pone.0197519.s004.pdf]

Table S3. Mantel tests at the county-level using downsampled data.

	**Phase correlations**	
	Correlation	*p*-value
Mantel tests		
Population*	−0.21	0.04
Distance	−0.13	0.08
Humidity	0.13	0.087
Temperature	−0.005	0.49
Region†	−0.66	0.0001
partial Mantel tests		
Population, adjusted for:		
Distance	−0.23	0.03
Humidity	−0.21	0.026
Temperature	−0.21	0.041
Region	−0.04	0.35
Distance, adjusted for:		
Population	−0.15	0.05
Humidity	−0.03	0.33
Temperature	−0.26	0.0006
Region	0.09	0.15
Humidity, adjusted for:		
Population	0.16	0.04
Distance	0.04	0.33
Temperature	0.20	0.01
Region	−0.11	0.11
Temperature, adjusted for:		
Population	0.009	0.43
Distance	−0.22	0.001
Humidity	−0.16	0.03
Region	−0.15	0.04
Region, adjusted for:		
Population	−0.64	0.0001
Distance	−0.66	0.0001
Humidity	−0.66	0.0001
Temperature	−0.67	0.0001

Mantel and partial Mantel tests using Spearman correlations to detect associations between the phase synchrony of Norwegian, Swedish, and Danish counties and a number of predictor variables (using downsampled Norwegian and Danish data).

* represents the product of population sizes for each municipality pair.
† binary variable indicating whether two counties are in the same country (0) or not (1).
