# Supplementary material for: Analysis of multi-level spatial data reveals strong synchrony in seasonal influenza epidemics across Norway, Sweden, and Denmark
Source: PLoS One. 2018 May 17;13(5):e0197519. doi: 10.1371/journal.pone.0197519 (PMC5957349; doi:10.1371/journal.pone.0197519)
Supplement: S4 Table — Mantel and partial Mantel tests using Spearman correlations to detect associations between the phase synchrony of Norwegian and Danish counties and a number of predictor variables (after excluding the Swedish data). (PDF) [file pone.0197519.s005.pdf]

**Table S4. Mantel tests at the county-level, excluding Sweden.**

| | Phase correlations | |
|---|---|---|
| | Correlation | $p$-value |
| *Mantel tests* | | |
| Population* | −0.30 | 0.04 |
| Distance | −0.13 | 0.17 |
| Humidity | 0.13 | 0.19 |
| Temperature | 0.04 | 0.36 |
| Region† | −0.69 | 0.0001 |
| *partial Mantel tests* | | |
| Population, adjusted for: | | |
| Distance | −0.31 | 0.04 |
| Humidity | −0.31 | 0.04 |
| Temperature | −0.31 | 0.04 |
| Region | 0.24 | 0.07 |
| Distance, adjusted for: | | |
| Population | −0.15 | 0.13 |
| Humidity | −0.03 | 0.40 |
| Temperature | −0.21 | 0.045 |
| Region | 0.13 | 0.20 |
| Humidity, adjusted for: | | |
| Population | 0.15 | 0.16 |
| Distance | 0.03 | 0.38 |
| Temperature | 0.17 | 0.12 |
| Region | −0.15 | 0.17 |
| Temperature, adjusted for: | | |
| Population | 0.06 | 0.31 |
| Distance | −0.17 | 0.09 |
| Humidity | −0.12 | 0.19 |
| Region | −0.13 | 0.19 |
| Region, adjusted for: | | |
| Population | −0.68 | 0.0001 |
| Distance | −0.69 | 0.0001 |
| Humidity | −0.69 | 0.0001 |
| Temperature | −0.70 | 0.0001 |

Mantel and partial Mantel tests using Spearman correlations to detect associations between the phase synchrony of Norwegian and Danish counties and a number of predictor variables (after excluding the Swedish data).

* represents the product of population sizes for each municipality pair.
† binary variable indicating whether two counties are in the same country (1) or not (0).
