# Supplementary material for: Analysis of multi-level spatial data reveals strong synchrony in seasonal influenza epidemics across Norway, Sweden, and Denmark
Source: PLoS One. 2018 May 17;13(5):e0197519. doi: 10.1371/journal.pone.0197519 (PMC5957349; doi:10.1371/journal.pone.0197519)
Supplement: S1 Fig — Municipality-level measures of population size (A), specific humidity (B), temperature (C), altitude (D), and airline travel (E). For details on how these data were obtained, see S1 Text. (PDF) [file pone.0197519.s006.pdf]

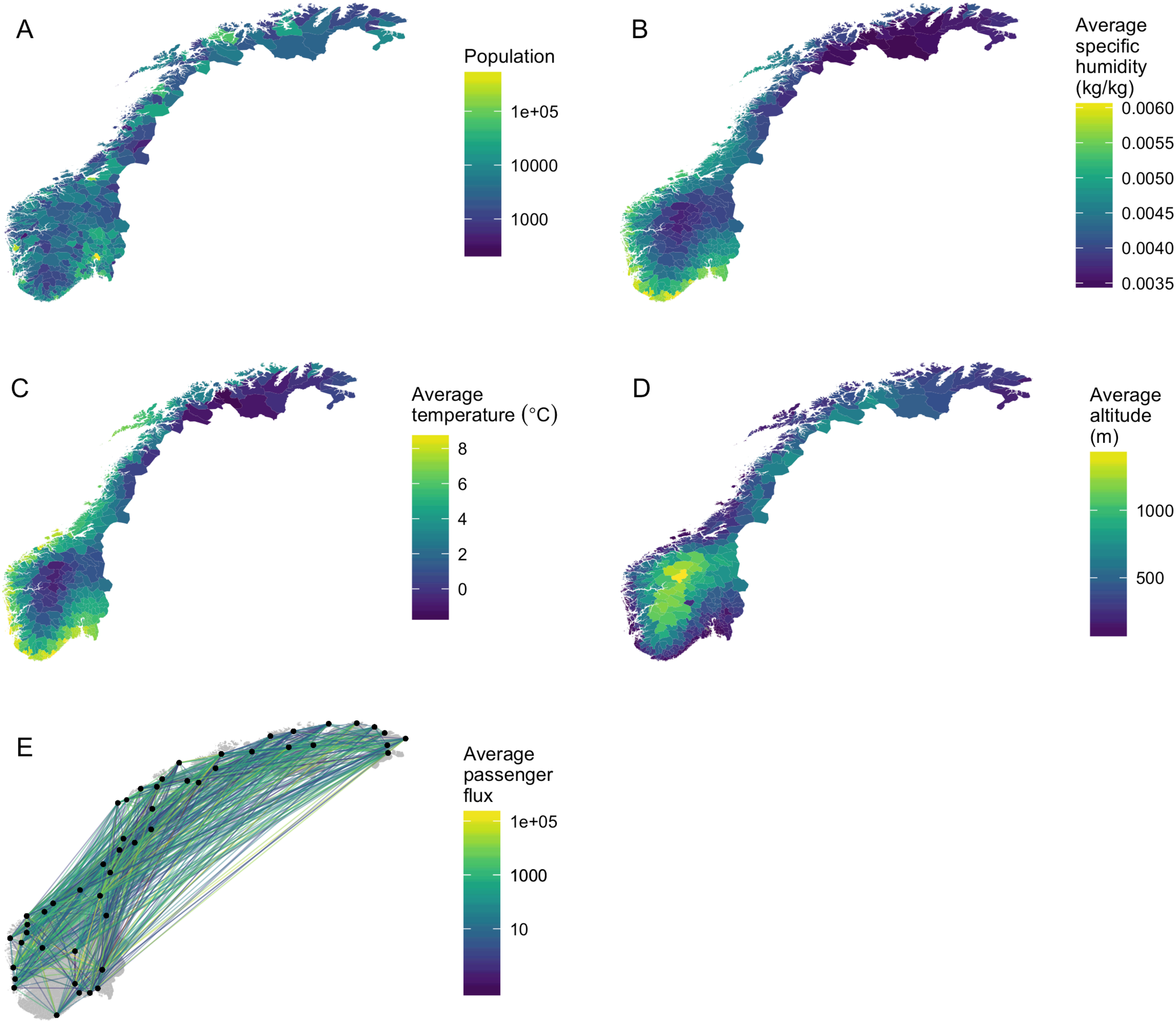

A
Population
1e+05
10000
1000
B
Average specific humidity (kg/kg)
0.0060
0.0055
0.0050
0.0045
0.0040
0.0035
C
Average temperature (°C)
8
6
4
2
0
D
Average altitude (m)
1000
500
E
Average passenger flux
1e+05
1000
10
