# Supplementary figures and images for: Analysis of multi-level spatial data reveals strong synchrony in seasonal influenza epidemics across Norway, Sweden, and Denmark
Source: PLoS One. 2018 May 17;13(5):e0197519. doi: 10.1371/journal.pone.0197519 (PMC5957349; doi:10.1371/journal.pone.0197519)

A

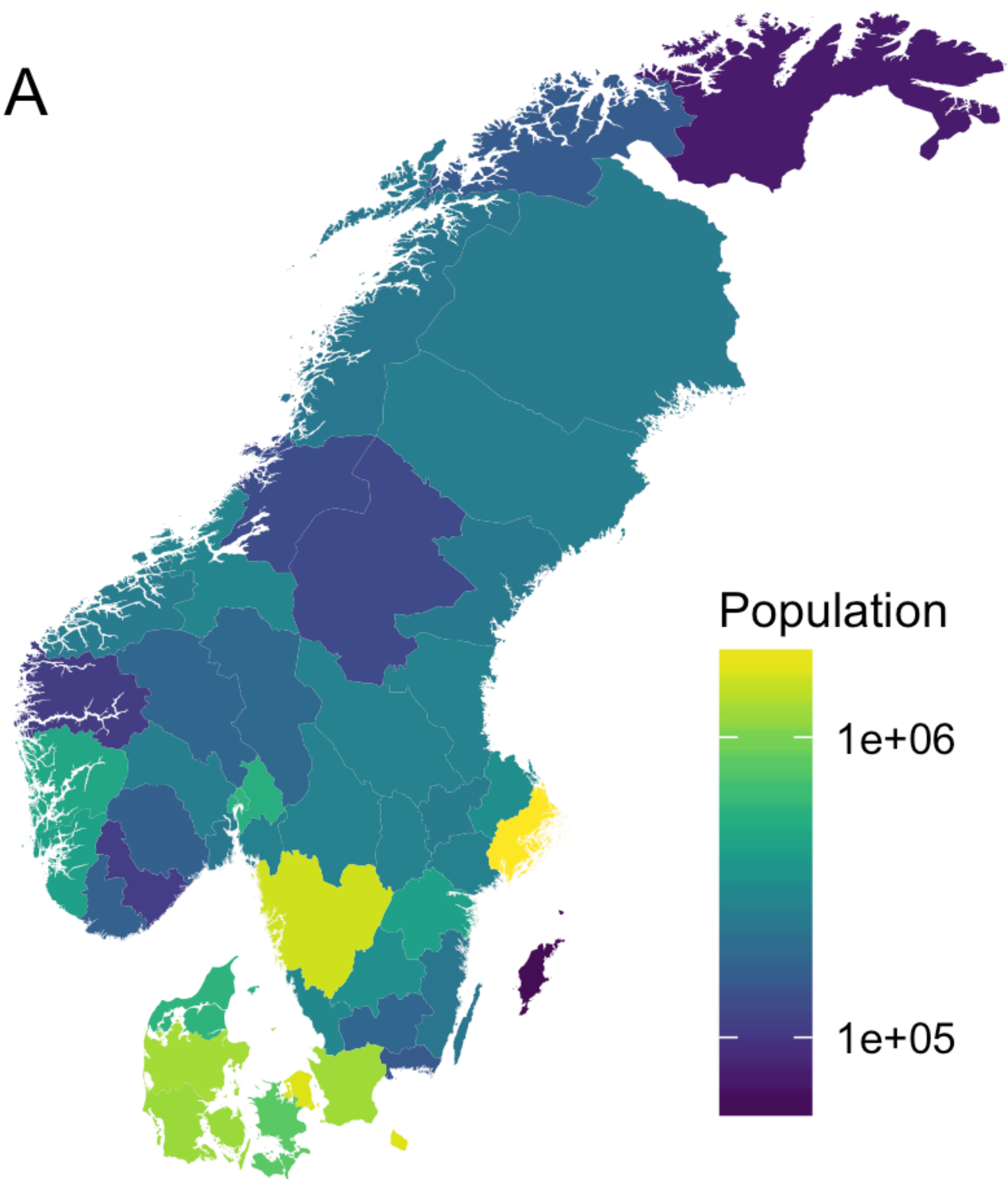

B

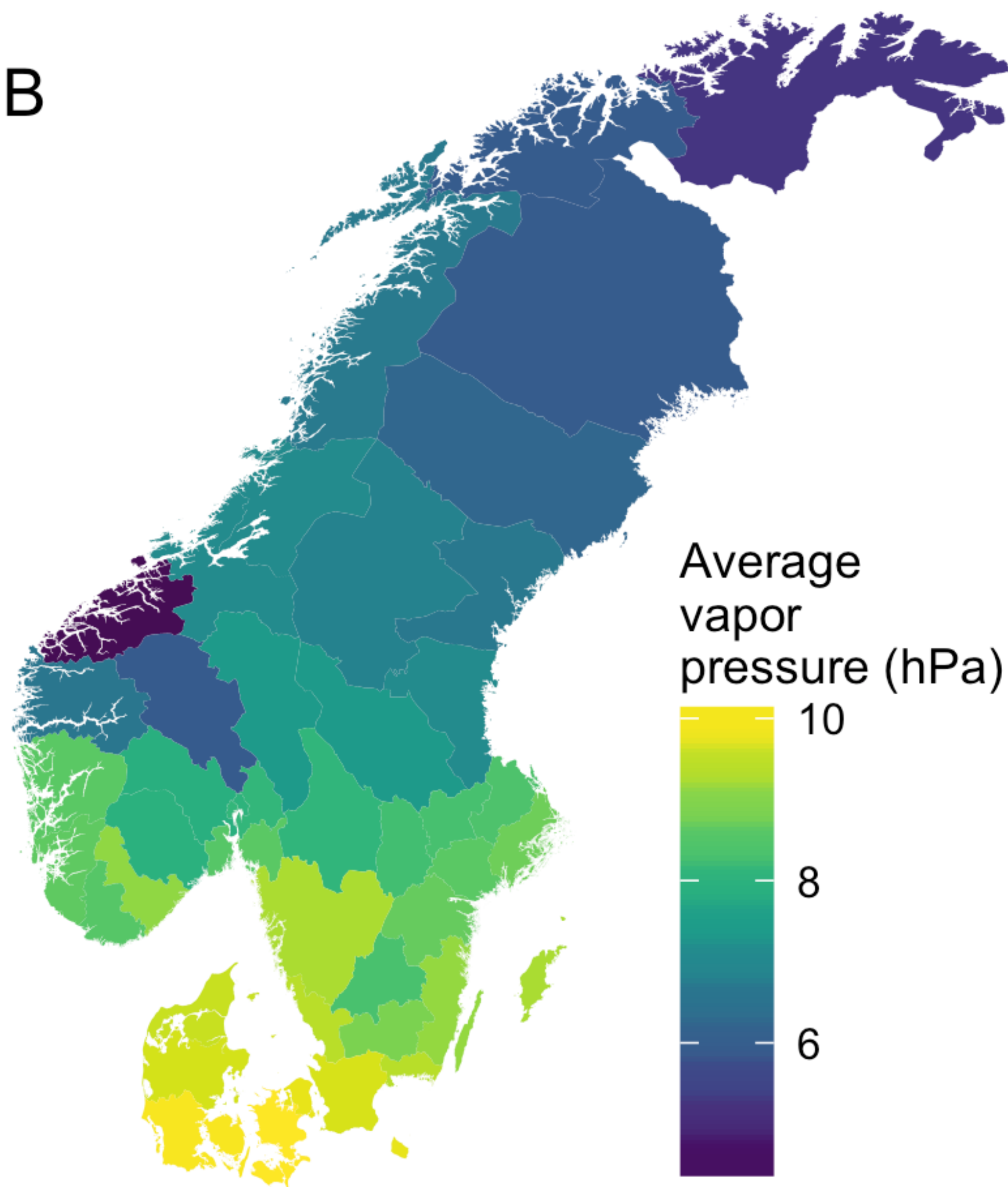

C

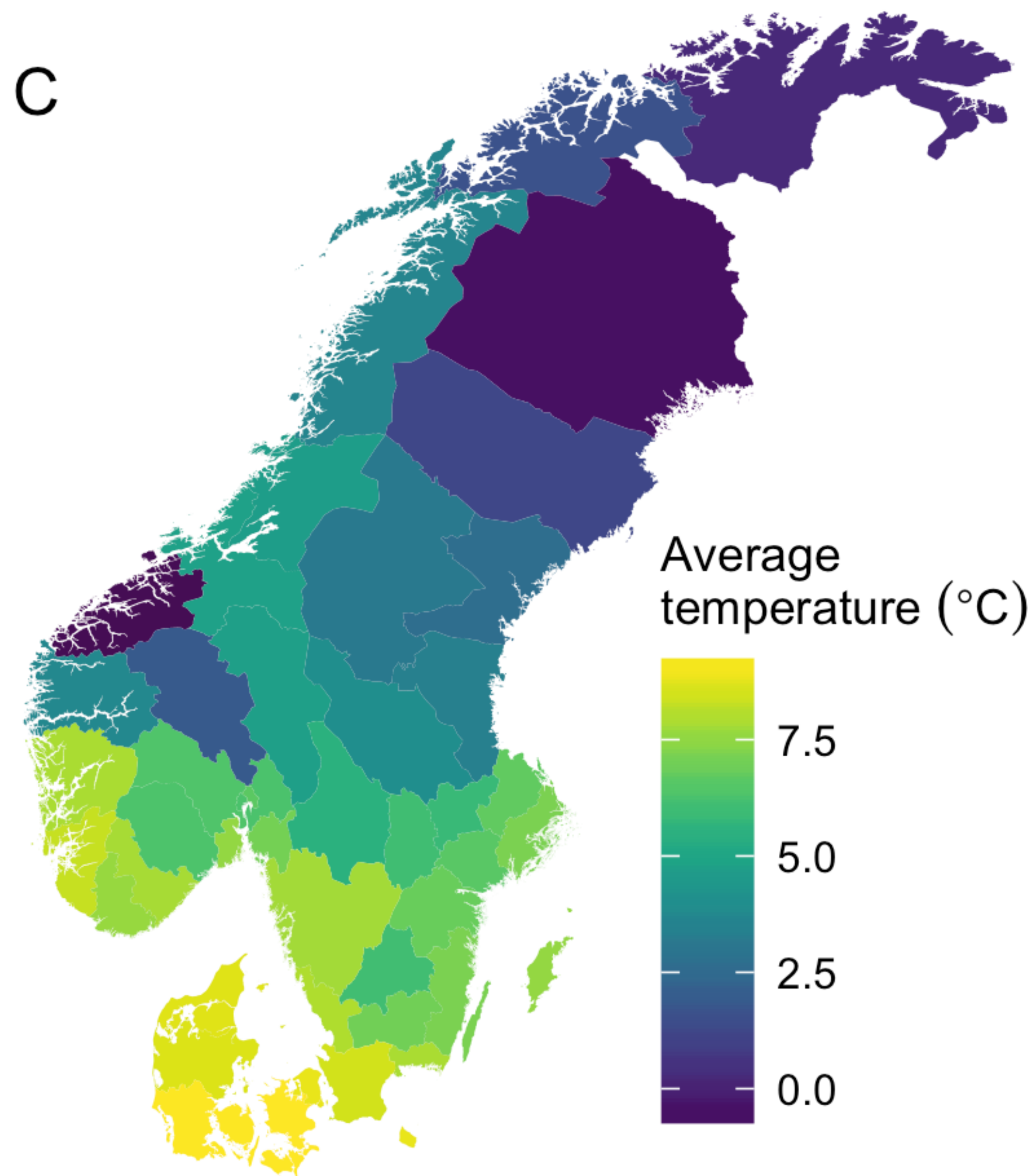

Supplement: S2 Fig — County-level measures of population size (A), vapor pressure (B), and temperature (C). For details on how these data were obtained, see S1 Text. (PDF) [file pone.0197519.s007.pdf]

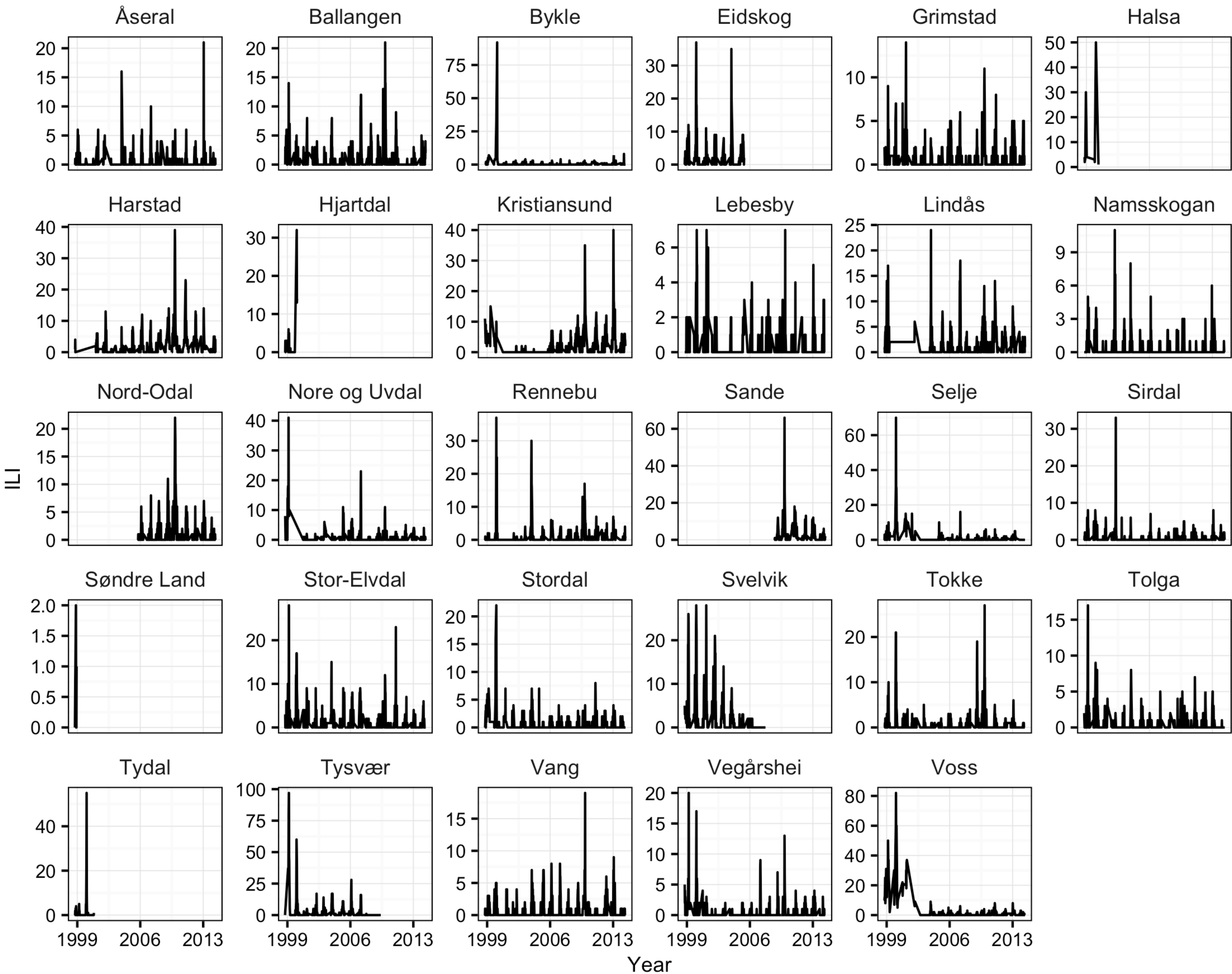

Supplement: S3 Fig — Norwegian ILI time-series that were removed from our analysis due to failure of a Box-Pierce white noise test or the presence of at least two seasons with less than ten cases reported. (PDF) [file pone.0197519.s008.pdf]

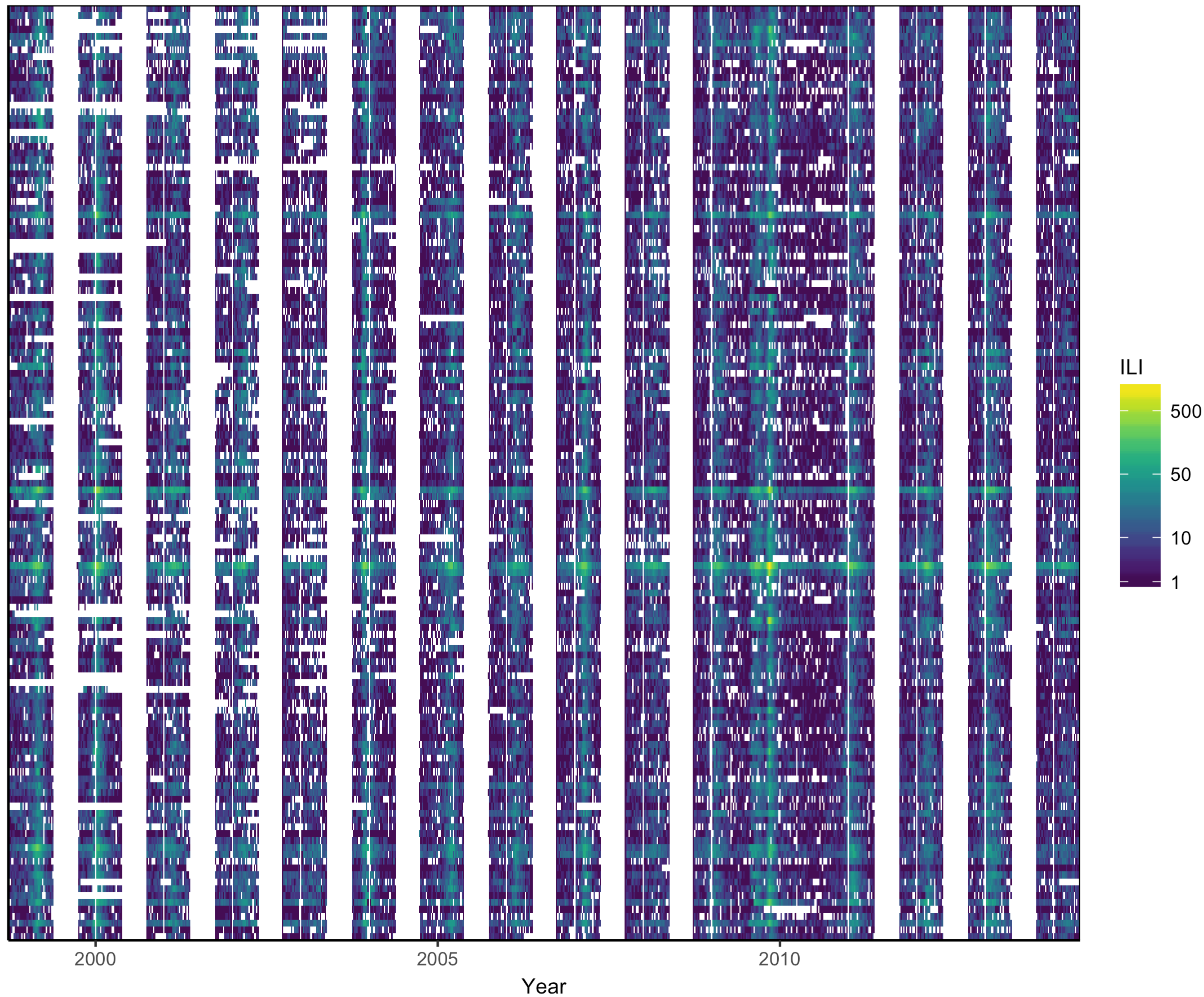

Supplement: S4 Fig — Norwegian ILI time-series that were used in all municipality-level analyses. Each row represents a distinct municipality, and these are ordered from top to bottom by decreasing latitude. Blank regions represent weeks for which we do not have data. (PDF) [file pone.0197519.s009.pdf]

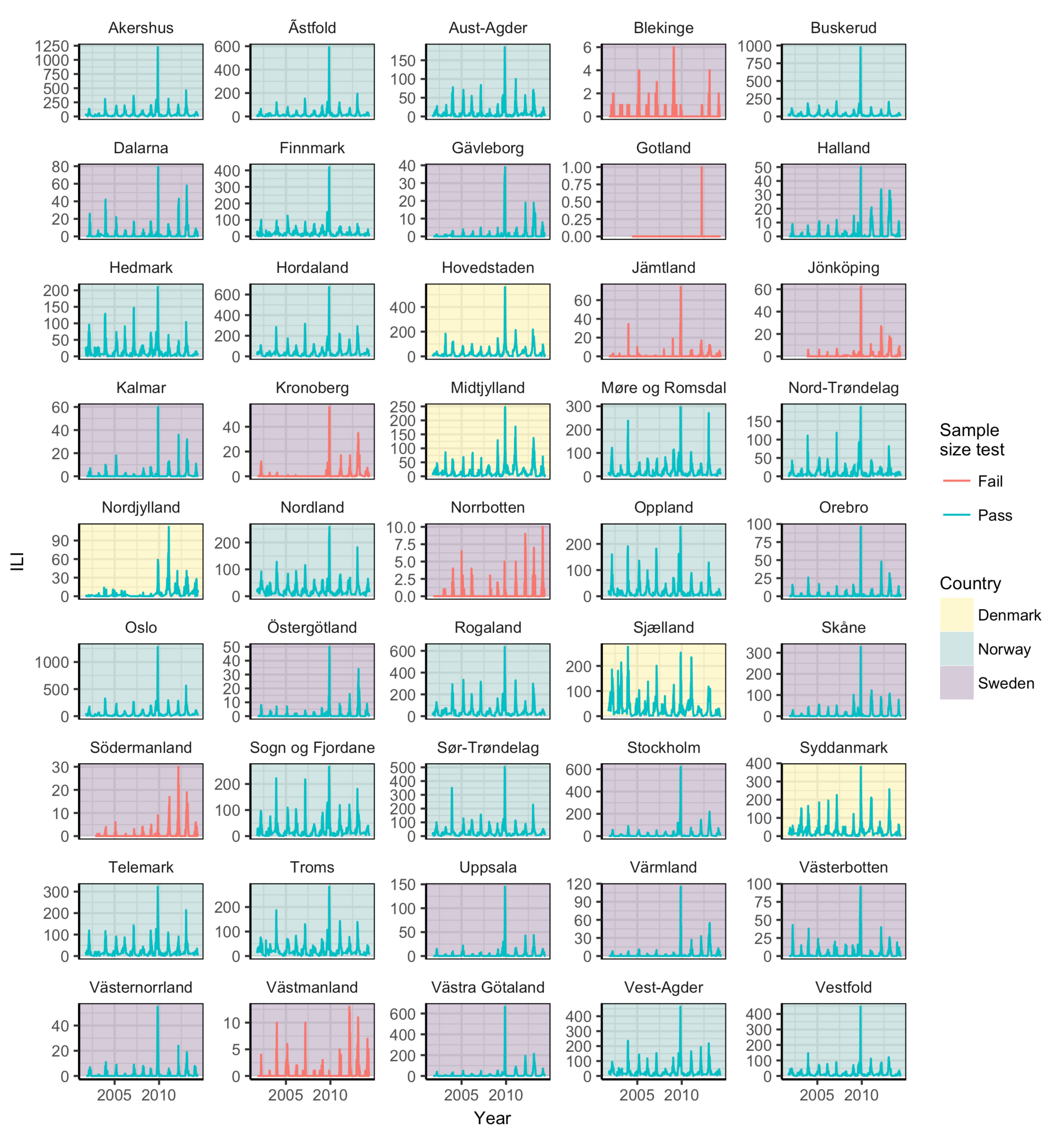

Supplement: S5 Fig — Norwegian, Swedish, and Danish county time-series. Red trajectories indicate that the data were removed from further analysis due to failure of a Box-Pierce white noise test or the presence of at least two seasons with less than ten cases reported; series that were kept for further analysis are depicted with blue trajectories. The background color of each plot indicates the country in which each county is located. (PDF) [file pone.0197519.s010.pdf]

Phase  
difference  
(days)

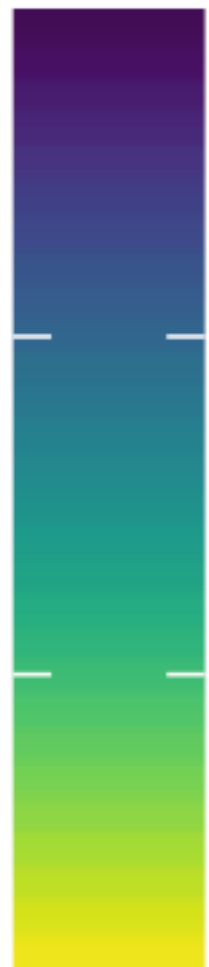

10

0

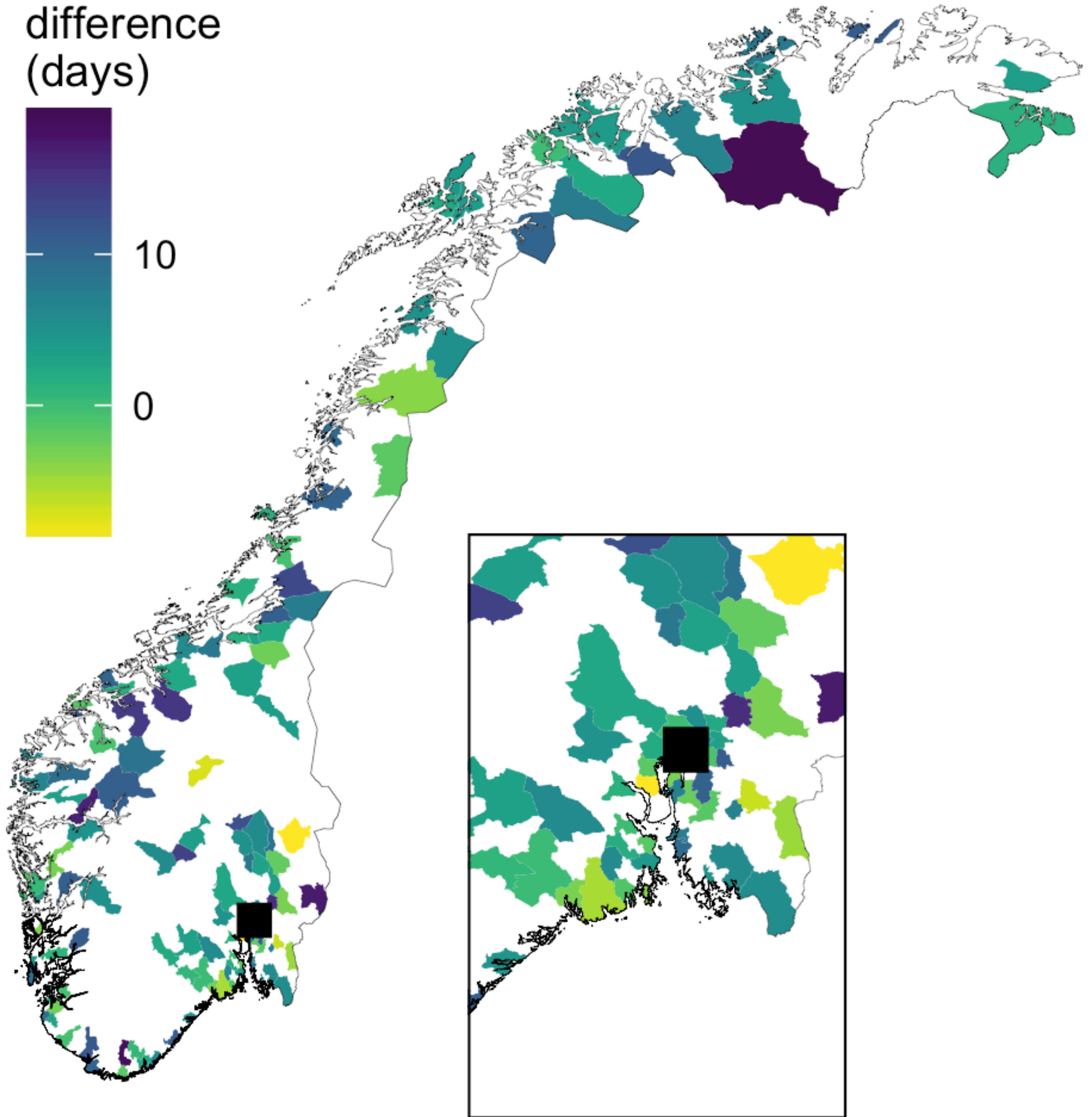

Supplement: S6 Fig — Average phase differences between each Norwegian municipality and Oslo. Oslo is indicated by the black square and the area surrounding the capital is enlarged in the inset box for clarity. A positive (negative) phase difference indicates epidemics tend to follow (precede) those in Oslo. (PDF) [file pone.0197519.s011.pdf]

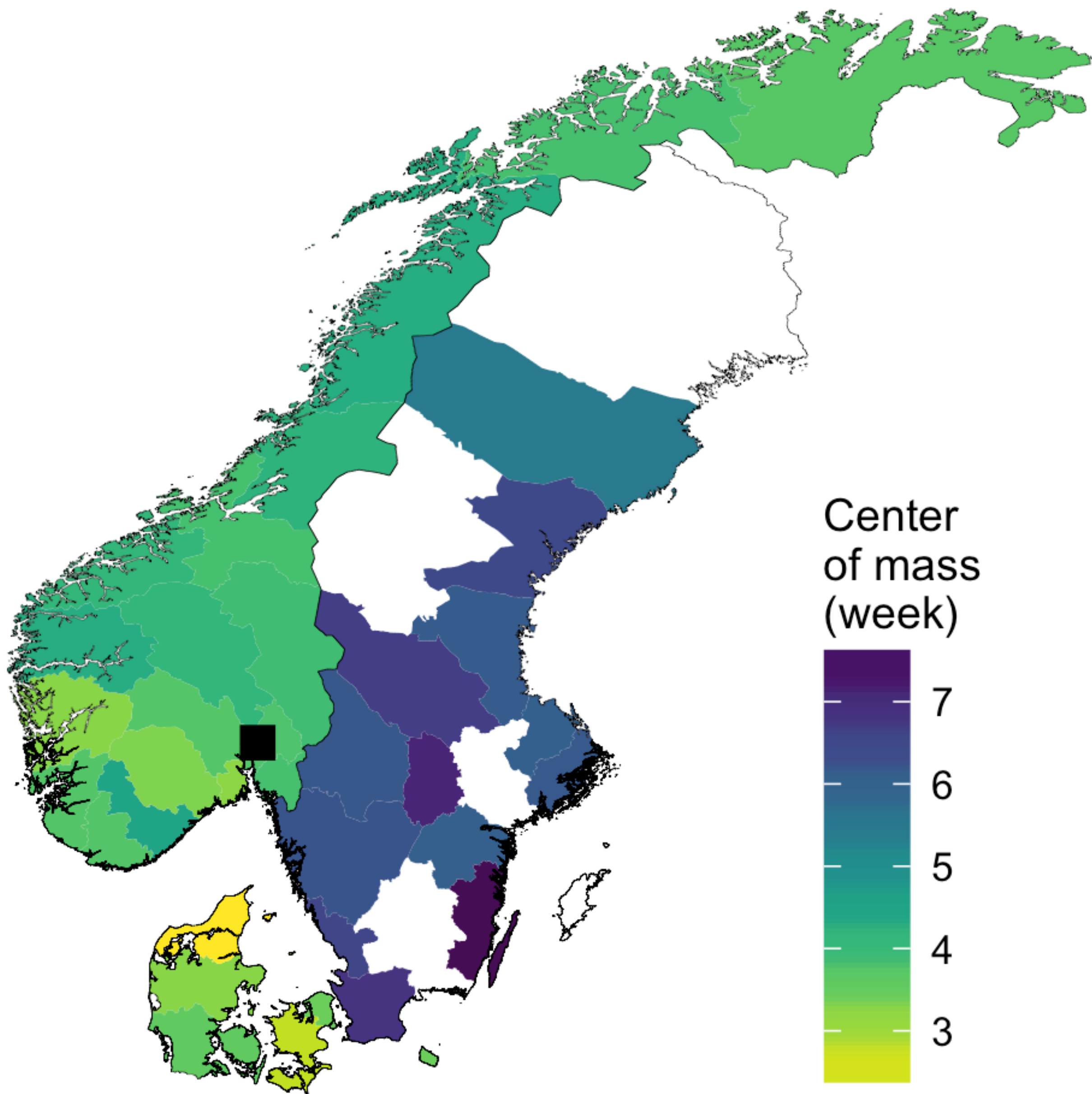

Center  
of mass  
(week)

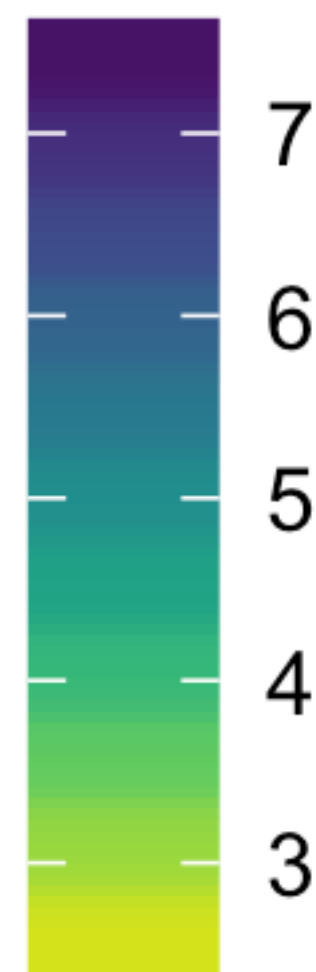

Supplement: S7 Fig — Colors indicate the average center of mass of epidemics (in calendar weeks) for each Norwegian, Swedish, and Danish county; counties for which data were discarded are shown in white. Oslo is indicated by the black square. (PDF) [file pone.0197519.s012.pdf]

A

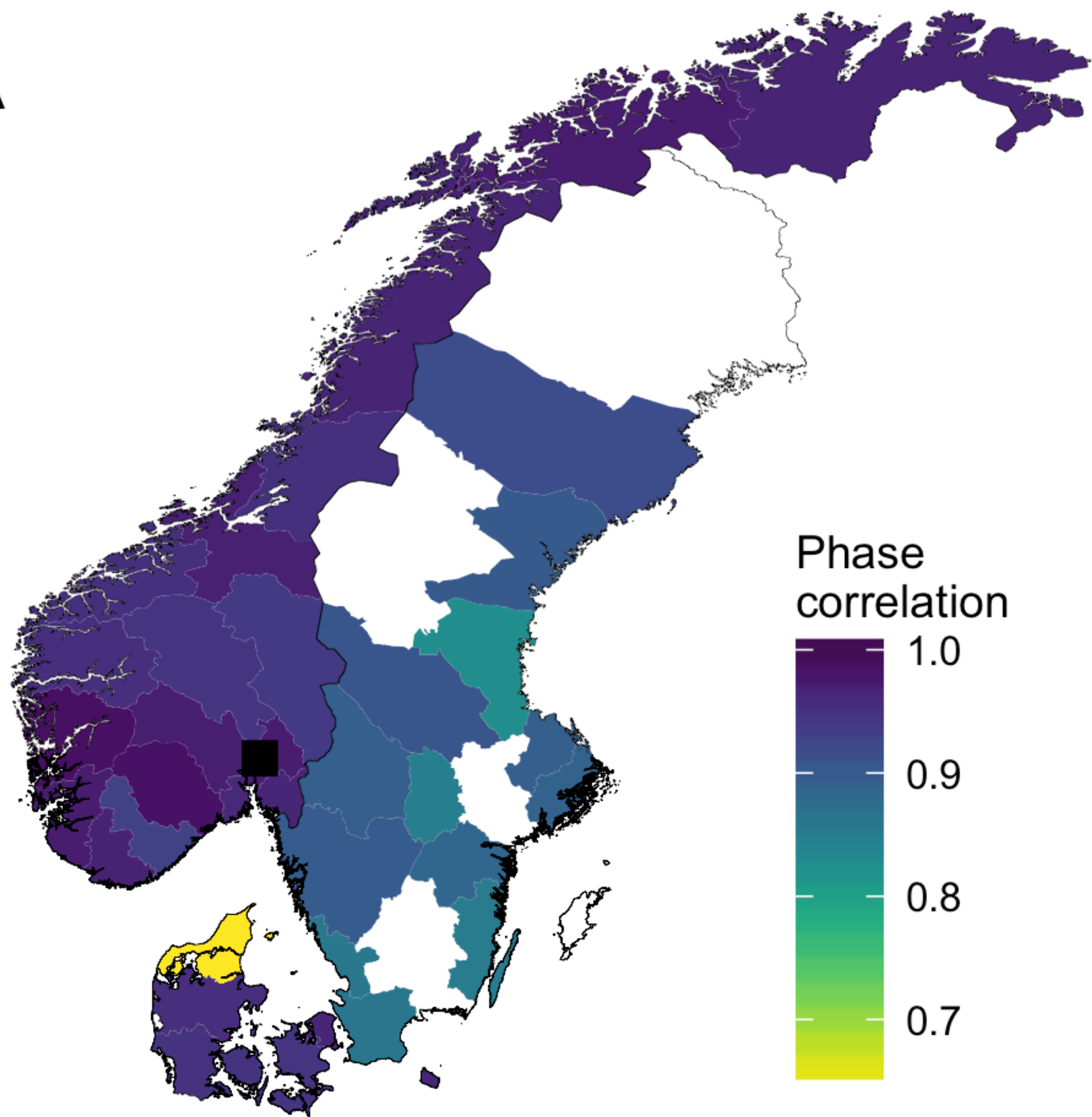

B

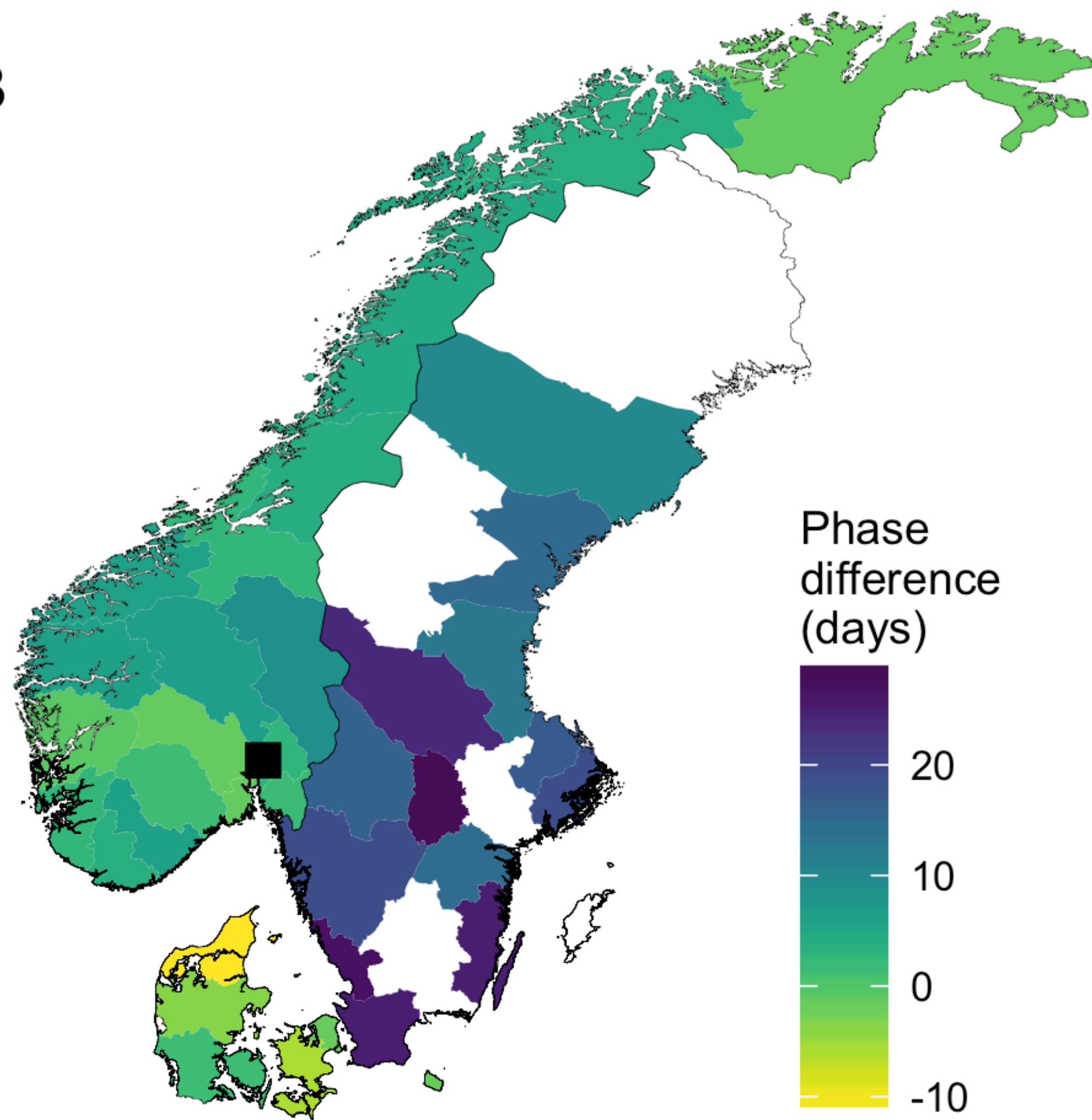

Supplement: S8 Fig — Colors indicate the correlation in phase-angles (A) and the average phase difference (B) between each county and Oslo (marked by the black square); counties for which data were discarded are shown in white. (PDF) [file pone.0197519.s013.pdf]

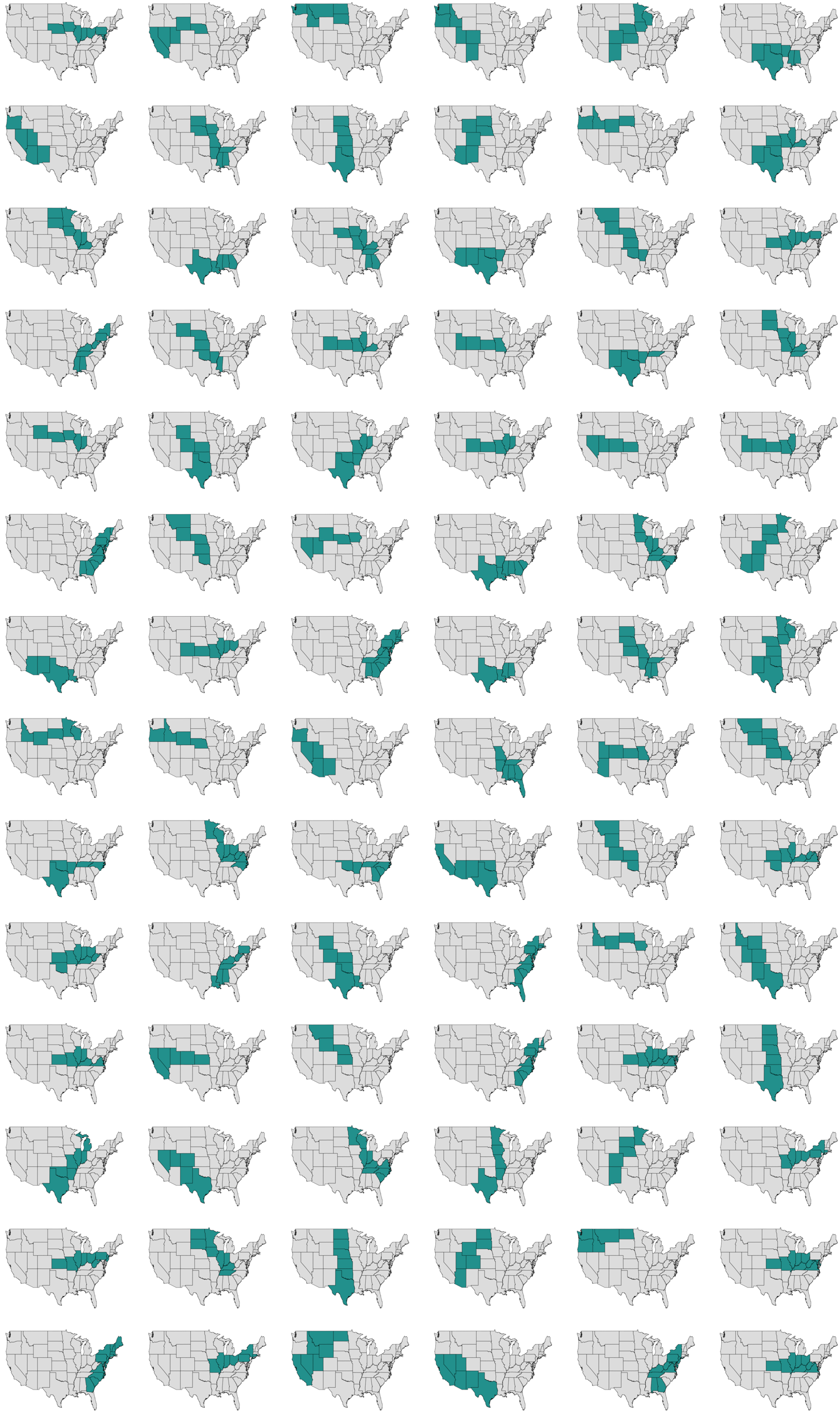

Supplement: S9 Fig — States in blue were those included in each transect. (PDF) [file pone.0197519.s014.pdf]

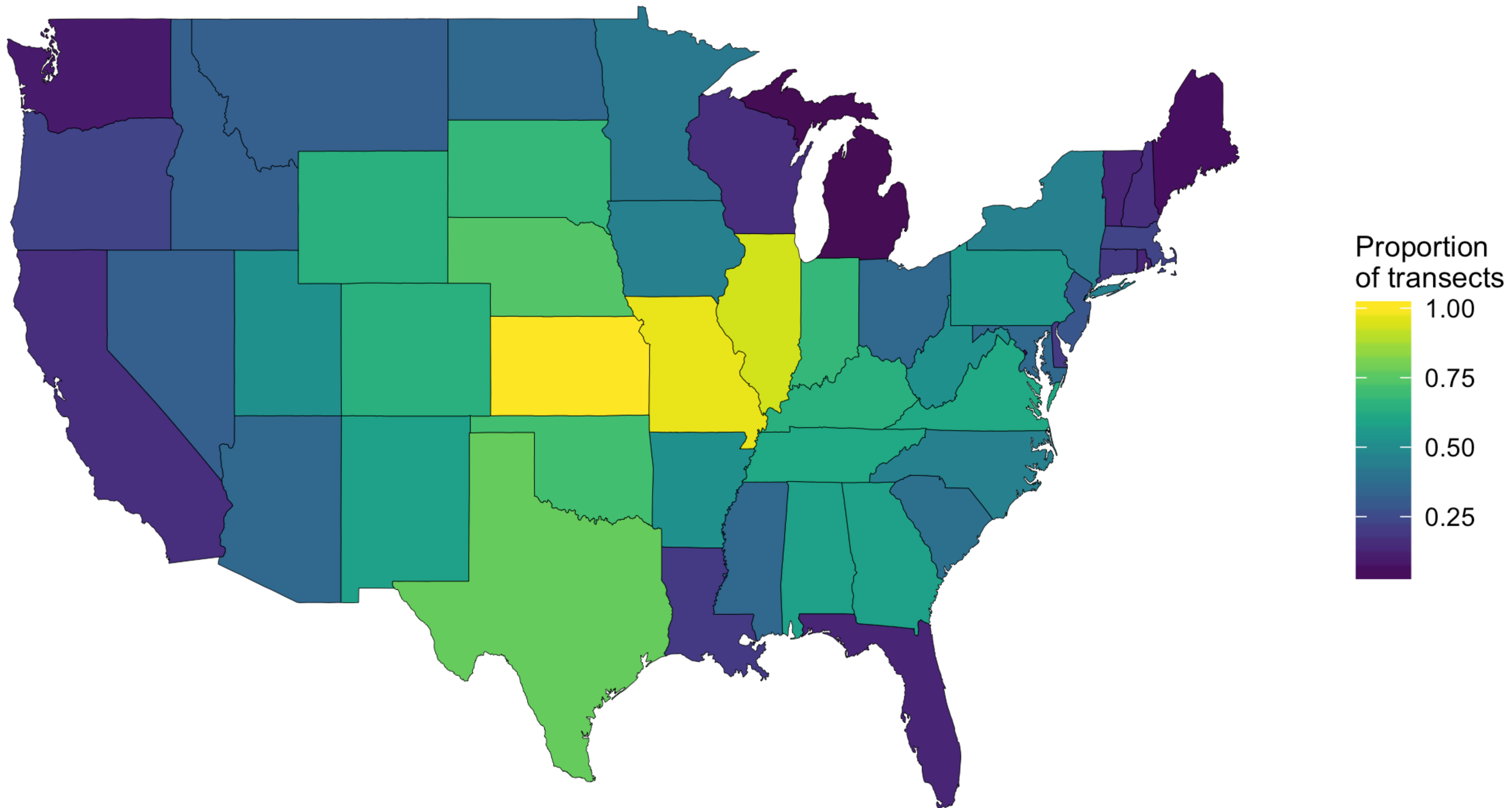

Supplement: S10 Fig — Each state is colored according to the proportion of transects in which it is included. (PDF) [file pone.0197519.s015.pdf]

A

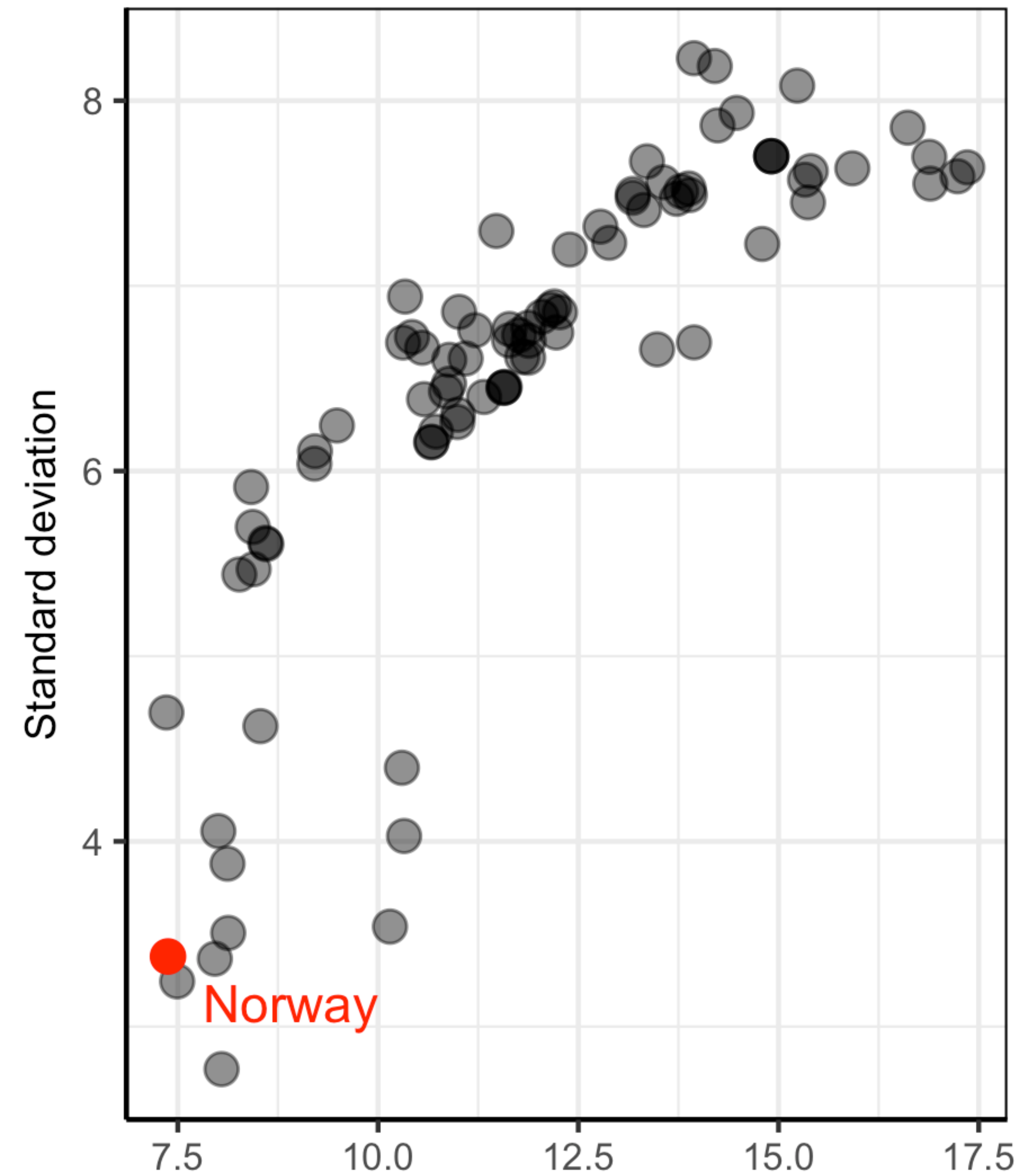

B

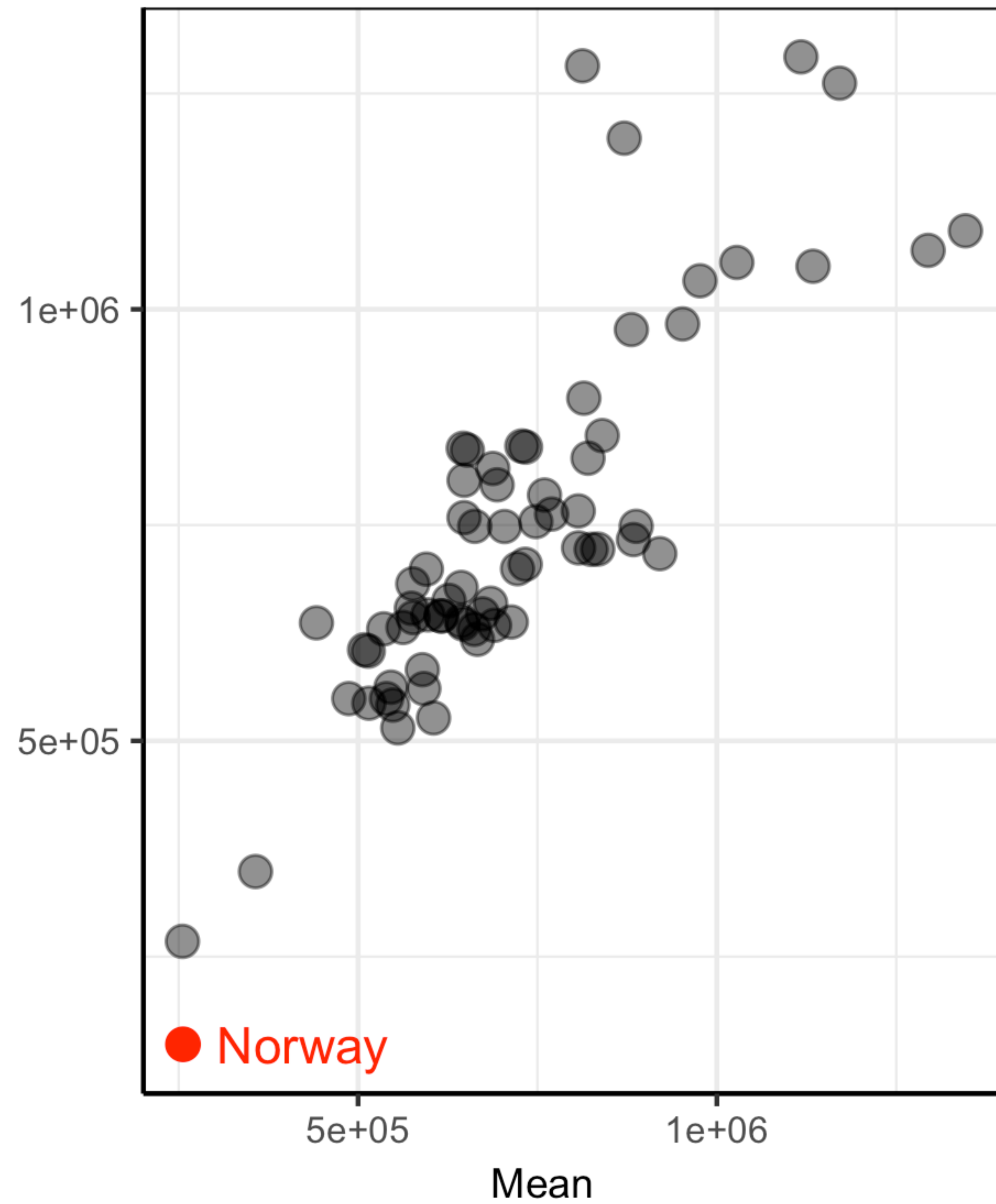

C

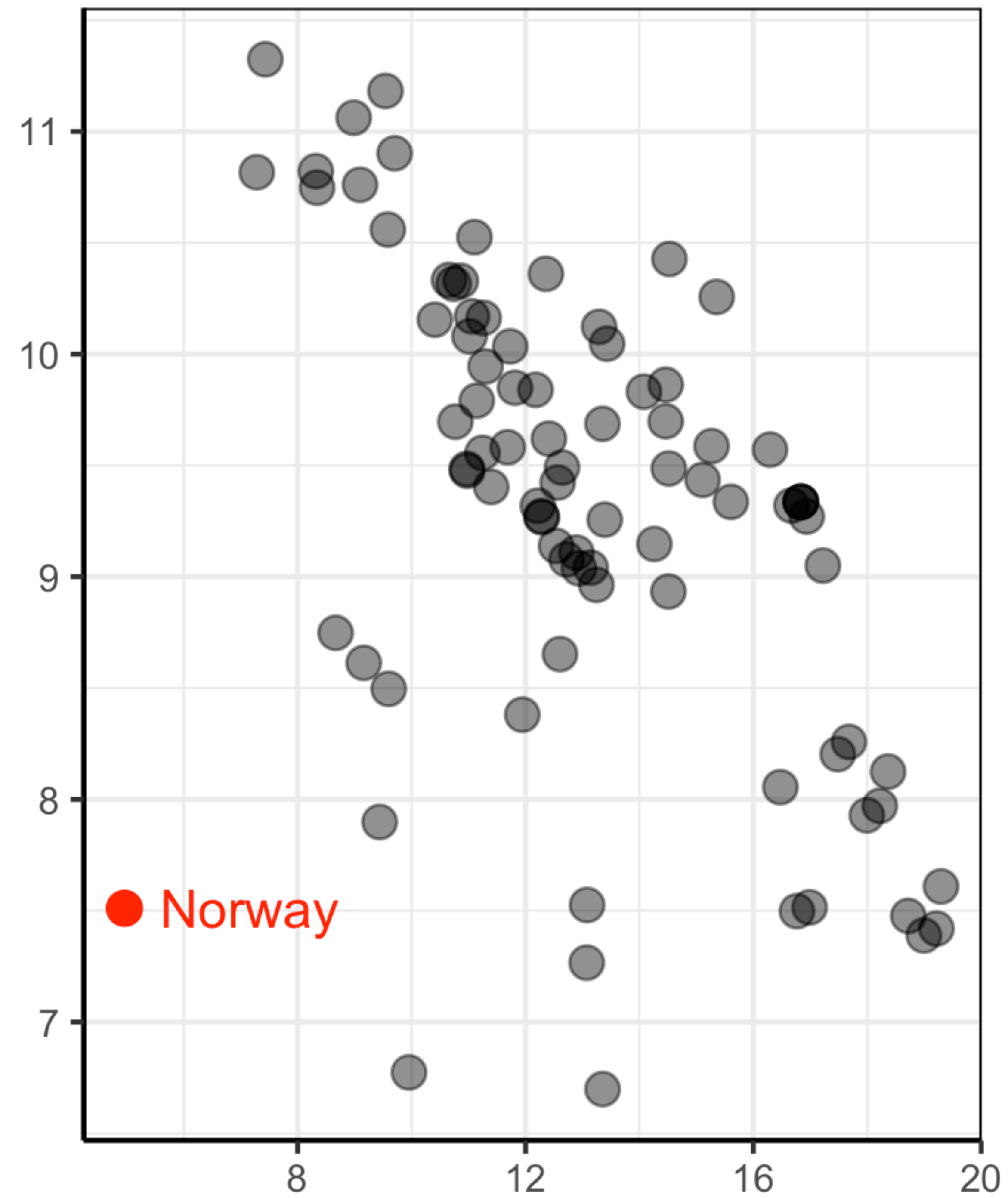

Supplement: S11 Fig — Mean and standard deviation of city humidity conditions (measured as vapor pressure) (A), population size (B), and temperature (C) within each US transect; red points indicate the corresponding values across Norwegian counties. (PDF) [file pone.0197519.s016.pdf]

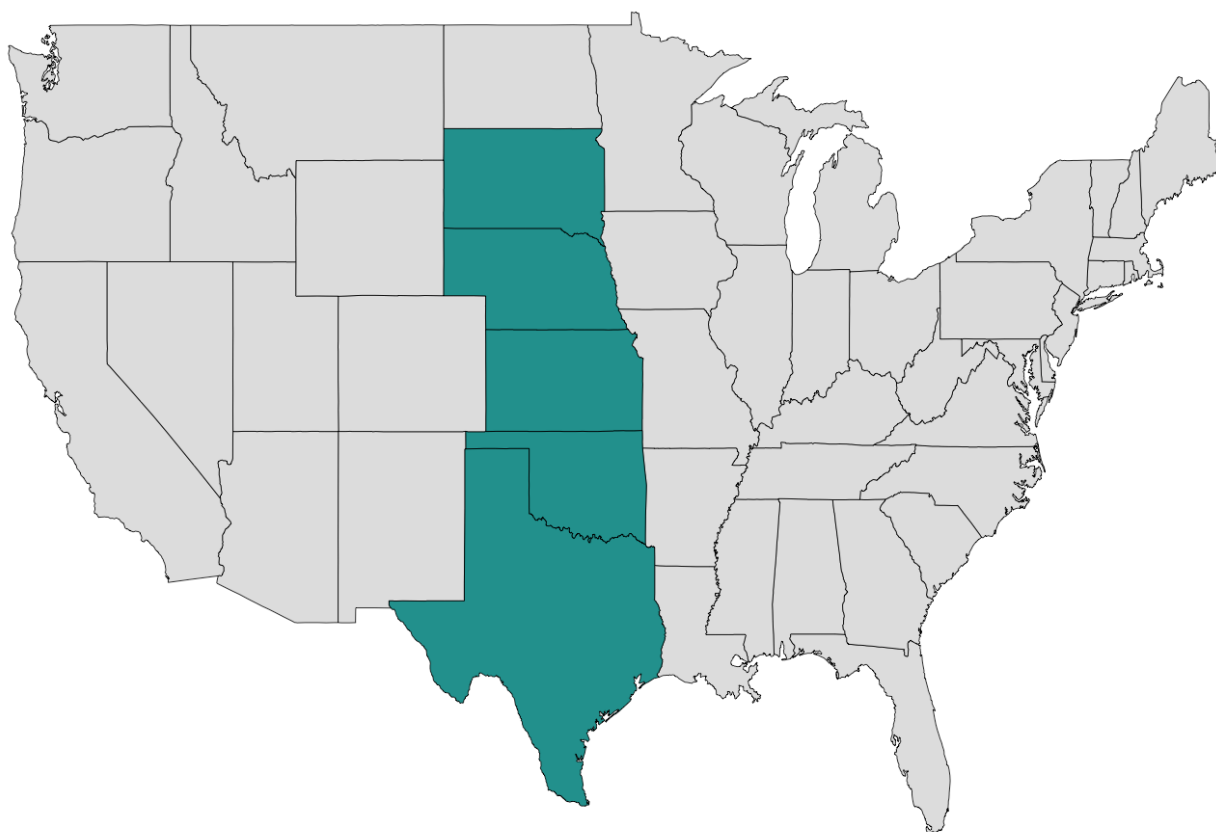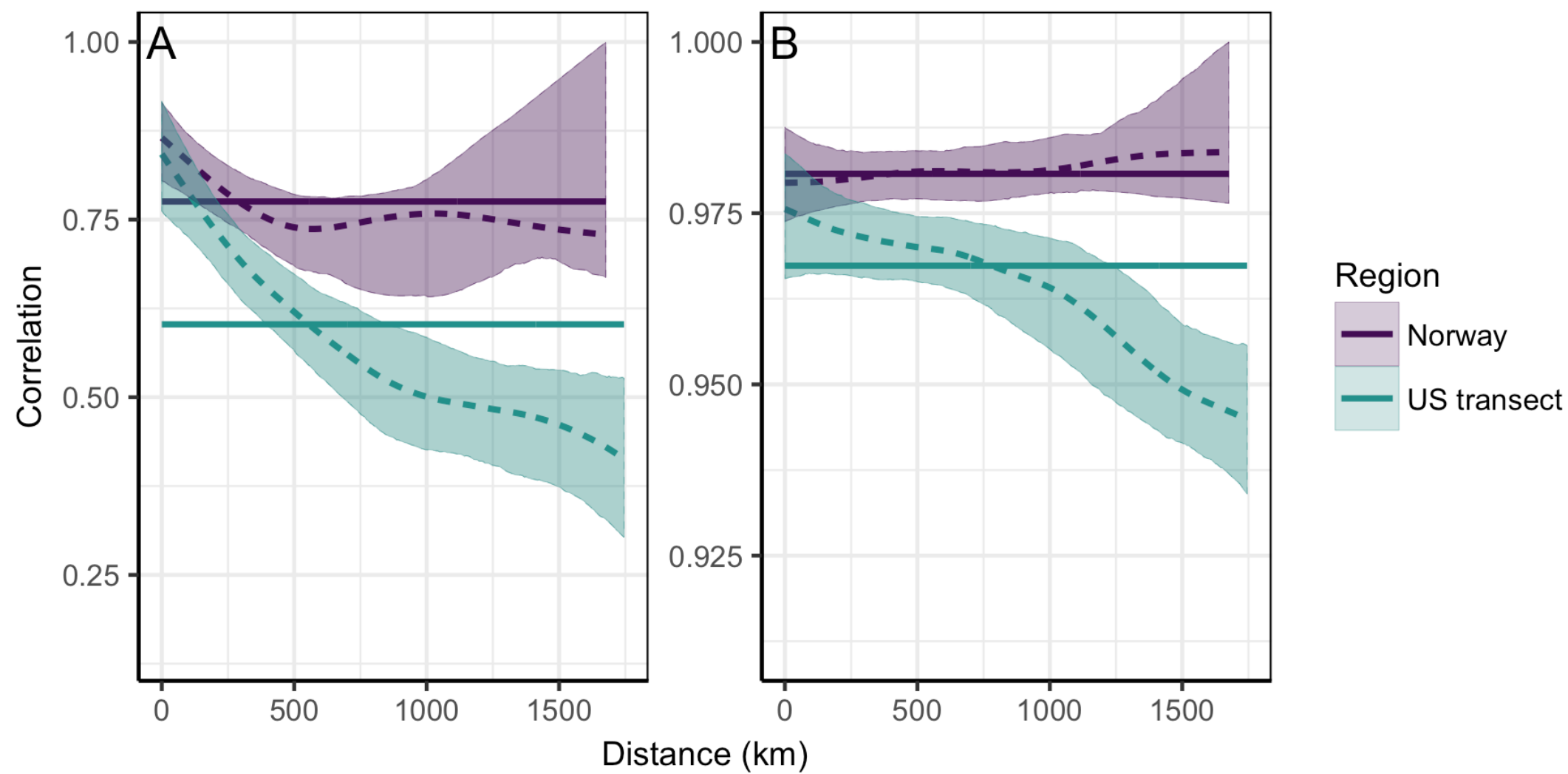

Supplement: S12 Fig — Top: blue states on the map were those included in the transect. Bottom: Spatial non-parametric correlation function comparing synchrony between the US transect (blue) and Norway (purple) for (A) ILI trajectories and (B) phase-angle trajectories. Solid lines represent the average synchrony across all regions, dashed lines depict the predicted relationship between synchrony and distance, and shaded regions are the 95% confidence intervals. (PDF) [file pone.0197519.s017.pdf]

A

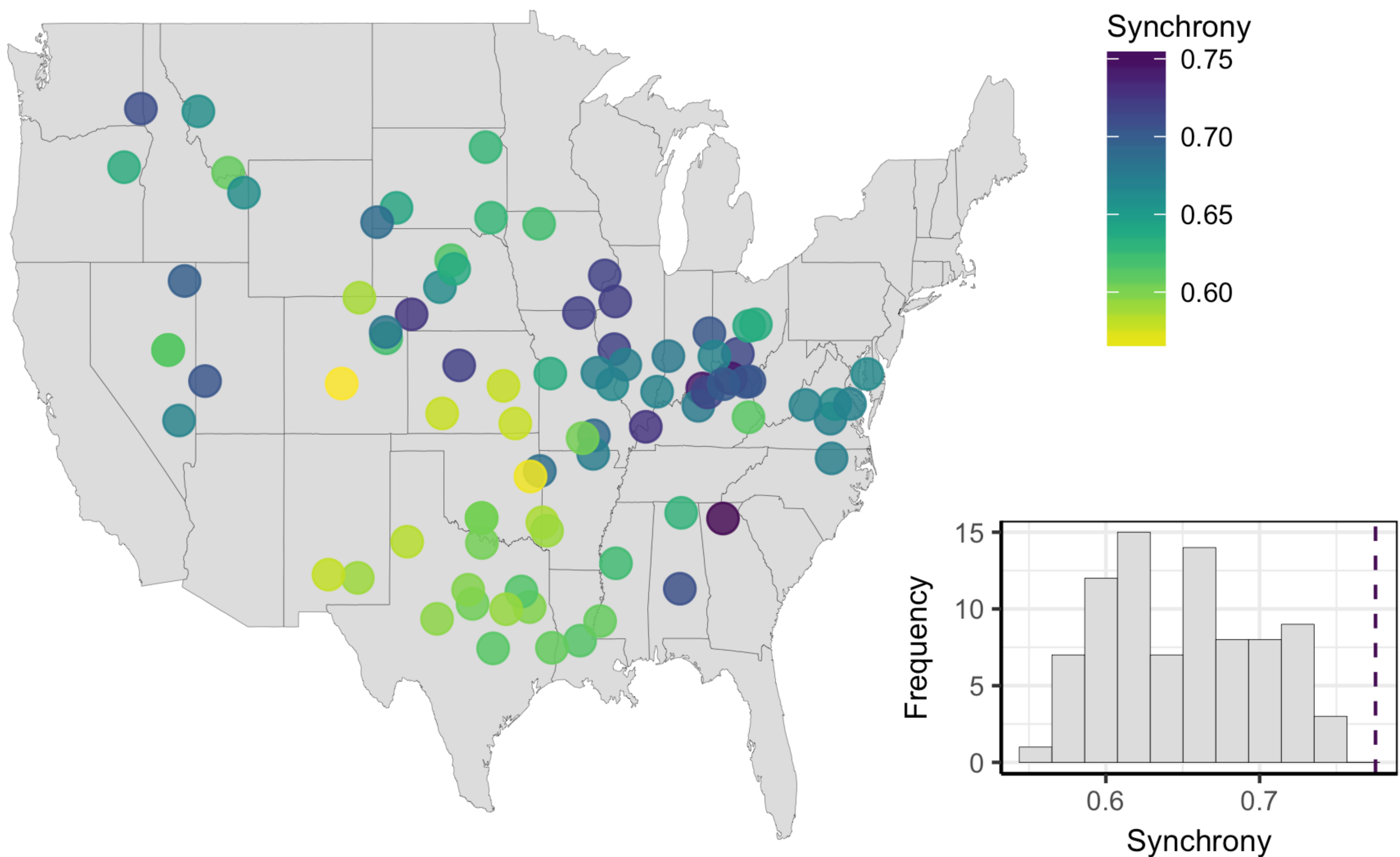

B

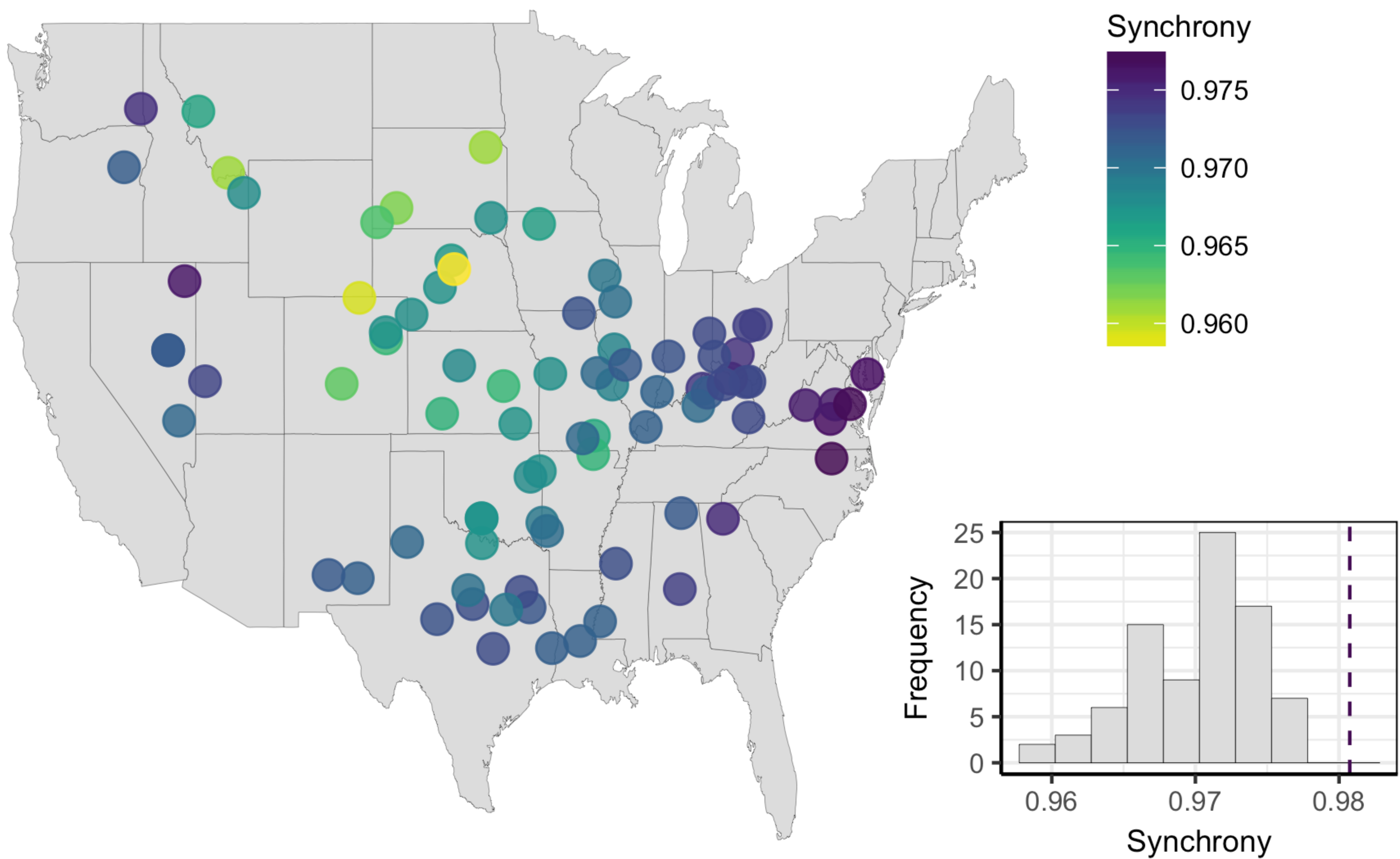

Supplement: S13 Fig — Average synchrony estimates for 84 US transects obtained by applying the spatial non-parametric correlation function to the abridged data of (A) ILI trajectories and (B) phase-angle trajectories. Points mark transect centroids and colors represent the corresponding synchrony estimate. Inset panels show the distribution of transect synchrony estimates; the estimate for Norway is marked by the dashed line. (PDF) [file pone.0197519.s018.pdf]

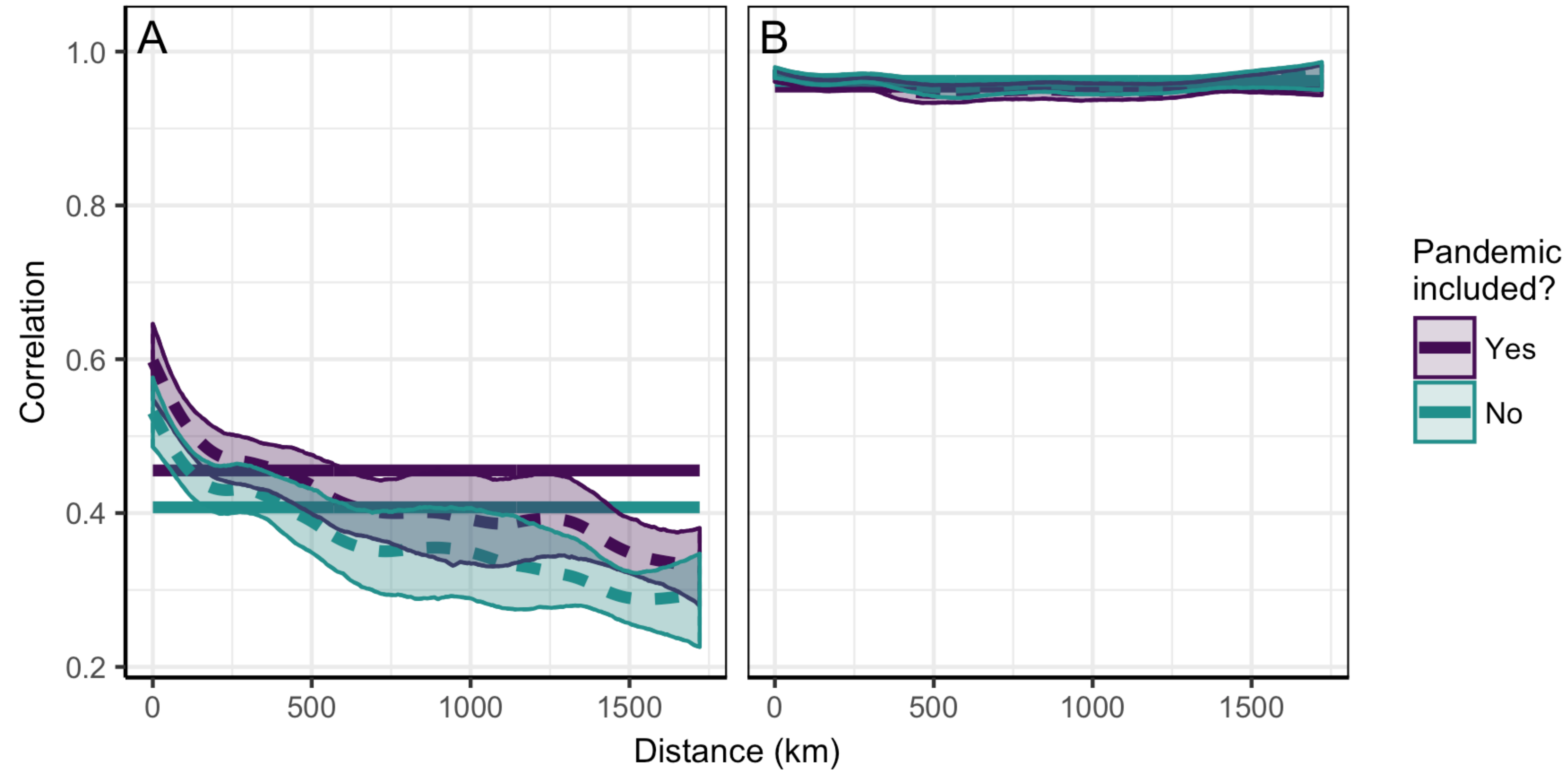

Supplement: S14 Fig — Spatial non-parametric correlation function for municipalities in Norway with (purple) and without (blue) the inclusion of the 2009/10 pandemic for (A) ILI trajectories and (B) phase-angle trajectories. Solid lines represent the average synchrony across all regions; dashed lines depict the predicted relationship between synchrony and distance; and shaded regions are the 95% confidence intervals. (PDF) [file pone.0197519.s019.pdf]
